# How Does the Tobacco Industry Attempt to Influence Marketing Regulations? A Systematic Review

**DOI:** 10.1371/journal.pone.0087389

**Published:** 2014-02-05

**Authors:** Emily Savell, Anna B. Gilmore, Gary Fooks

**Affiliations:** 1 Department for Health, University of Bath, Bath, United Kingdom; 2 UK Centre for Tobacco and Alcohol Studies, Bath, United Kingdom; Brunel University, United Kingdom

## Abstract

**Background:**

The Framework Convention on Tobacco Control makes a number of recommendations aimed at restricting the marketing of tobacco products. Tobacco industry political activity has been identified as an obstacle to Parties’ development and implementation of these provisions. This study systematically reviews the existing literature on tobacco industry efforts to influence marketing regulations and develops taxonomies of 1) industry strategies and tactics and 2) industry frames and arguments.

**Methods:**

Searches were conducted between April-July 2011, and updated in March 2013. Articles were included if they made reference to tobacco industry efforts to influence marketing regulations; supported claims with verifiable evidence; were written in English; and concerned the period 1990–2013. 48 articles met the review criteria. Narrative synthesis was used to combine the evidence.

**Results:**

56% of articles focused on activity in North America, Europe or Australasia, the rest focusing on Asia (17%), South America, Africa or transnational activity. Six main political strategies and four main frames were identified. The tobacco industry frequently claims that the proposed policy will have negative unintended consequences, that there are legal barriers to regulation, and that the regulation is unnecessary because, for example, industry does not market to youth or adheres to a voluntary code. The industry primarily conveys these arguments through direct and indirect lobbying, the promotion of voluntary codes and alternative policies, and the formation of alliances with other industrial sectors. The majority of tactics and arguments were used in multiple jurisdictions.

**Conclusions:**

Tobacco industry political activity is far more diverse than suggested by existing taxonomies of corporate political activity. Tactics and arguments are repeated across jurisdictions, suggesting that the taxonomies of industry tactics and arguments developed in this paper are generalisable to multiple jurisdictions and can be used to predict industry activity.

## Background

The public availability of internal tobacco industry (TI) documents resulting from state-level litigation and the signing of the Master Settlement Agreement (MSA) in the USA has formed the basis of an extensive body of work on TI political activity (see [2] for overview). These studies have greatly expanded our understanding of the scope of TI political activity, but they tend to be event or case-study based. While the focused nature of these studies provides potentially valuable detail of the political strategies used by large tobacco companies, they do not draw out the broader trends and patterns of TI political activity, and with almost 800 publications now based on these documents [3] it is increasingly difficult for public health advocates and policymakers to learn from the research findings. Only two studies have reviewed elements of this literature systematically [1], [4], and none have attempted to develop taxonomies where industry tactics and arguments can be assessed and systematically categorised in a way that could be applied to other areas of public health involving corporate interests.

This paper therefore aims to both systematically review the existing literature on strategies used by the TI to influence regulation aimed at restricting the marketing of tobacco products, and to develop taxonomies for categorising the tactics and arguments used. By providing a summary of industry actions in this area, this review is likely to be a valuable resource for enhancing the ability of public health advocates and policymakers to understand, predict, and potentially counter tactics the TI might use to exert influence on policy and the types of arguments it is most likely to make when it does. This is particularly important given that multiple Articles of the World Health Organisation’s Framework Convention on Tobacco Control (FCTC) make recommendations regarding the marketing of tobacco products, for example Article 13 recommends a “comprehensive ban on advertising, promotion and sponsorship” [5]. The FCTC covers 87.4% of the world’s population [6], but despite the vast majority of states becoming Party to the FCTC many have yet to implement its recommendations [7] with the tactics of the TI identified as a hindrance to the development and implementation of legislation [8]. Existing research shows that despite TI claims that marketing is only used for brand switching and capturing market share, there is a significant link between TI marketing and smoking initiation among young people and increased smoking prevalence [9], [10], [11], [12], [13], [14], [15]. This underpins the continuing importance of understanding the strategies the TI use to shape policies aimed at regulating the marketing of tobacco products which kill half of their long-term users [16], [17].

## Methods

This review aimed to identify all articles that examined TI attempts to influence marketing regulation from 1990 to 2013 (see Protocol S1). Marketing encompasses five key variables: product, promotion, price, place, and person [18]. However as a systematic review of TI influence on tobacco tax has already been completed [1], we excluded price in the form of tax from this review.

The databases Web of Knowledge (which includes Web of Science, BIOSIS Previews, and MEDLINE), Business Source Premier, and Embase were searched using the search string: (corporat* OR industr* OR compan* OR busines* OR firm*) AND (tobacco OR smok* OR cigarette*) AND (marketing OR advertis* OR sponsor*) AND (regulat* OR policy OR legislat*). The search engine Google was used to identify grey literature, the UCSF Tobacco Documents ‘Marketing and Advertising’ Bibliography [19] was searched for additional academic articles, the series of UCSF US State tobacco reports [20] were assessed, and experts were contacted to identify any additional papers (more information is available in Appendix S1). All searches were conducted between April and July 2011, and were updated in March 2013. Searches were limited to articles from 1990 to 2013 and those written in English. The search protocol was developed in conjunction with a qualified librarian.

Initial study inclusion/exclusion criteria were discussed extensively between all three authors, piloted and re-piloted. The final inclusion/exclusion criteria used in this review can be seen in Box S1. In total 1754 articles were identified, of which 1326 were excluded based on their title and abstract alone. 418 articles were downloaded for full analysis (10 articles could not be located despite efforts to contact the authors). 370 articles were excluded for not meeting the inclusion criteria. The remaining 48 articles met all of the inclusion criteria.

Data extraction (Appendix S1 provides a summary) was undertaken by the lead author, and a random sample of 24 (50%) of the included articles were second-reviewed by both the second and third authors to check that all the inclusion criteria were met and to agree final tactic and argument categorisation. All differences were discussed between all three authors. Disagreements related only to categorisation, more often in relation to the categorisation of arguments than tactics. Where disagreement occurred, all evidence falling under that particular category was re-reviewed by all three authors until agreement had been reached. Narrative synthesis was undertaken to combine the evidence from the articles.

### Taxonomies

This review splits TI political activity into ‘strategies’ which include individual ‘tactics’ (the methods by which a corporation attempts to exert influence) and ‘frames’ which include individual ‘arguments’ (the reasons given by a corporation as to why they oppose one idea or support another).

Hillman and Hitt’s (1999) paper [21] was used as the basis for the initial categorisation of TI strategies/tactics as it is the most widely cited attempt to analytically categorise the tactics used by corporations attempting to influence policy. Their system of classification, based on resource dependence and market exchange theory, assumes that corporate political activity represents one side of an exchange relationship in which corporations offer policymakers support and information in return for influencing policy. They identify three ‘long-term’ political strategies (information strategy, financial incentive strategy, and constituency-building strategy) which each contain multiple ‘short-term’ tactics. While they claim their list is a “comprehensive taxonomy of specific political strategies” [21], it preceded the TI document literature which, given the uniqueness of the resource, is arguably the richest literature available on industry political influence. Perhaps unsurprisingly, therefore, we identified additional strategies and tactics that were not included within their categorisation.

Hillman and Hitt’s [21] categorisation did not consider the frames or arguments used by Industry, and so we developed a second categorisation to take account of this. Frames offer a way of “packaging” an issue [22], they provide a “summary message for a defined topic area” [23] and may contain several arguments that share a common perspective [22], [23]; in other words, frames are the “meta-message” [24]. Many papers, within tobacco [22], [23], [25], [26], [27], [28], [29], [30] and other areas [31], [32], [33], [34], have shown that how an argument or issue is framed is important for its success and how it is perceived. It was therefore deemed important for this review to categorise both the arguments the TI uses, and the broader frames in which they fit. There is no consensus in the literature about the naming of frames.

We initially developed our list of strategies/tactics and frames/arguments via ‘a priori coding’ [35], the former adapted from Hillman and Hitt [21] and the latter based on the limited existing literature on TI frames [26], [28]. Additional categories were added via ‘emergent coding’ [35] following review of the papers included and after extensive discussions between all three authors. This was an iterative process and the taxonomies were only finalised after all of the papers had been reviewed as described above (detailed descriptions of the strategies and frames are available in Appendix S1).

### Categorisation

In this paper the tactics and arguments used by the TI were categorised using the taxonomies outlined above. We then counted the number of times each was used. If a tactic or argument was referred to more than once (in one or multiple articles) regarding the *same* policy then it was only counted once, however if it was referred to more than once about *different* policies then this was counted separately. While the tactics and arguments counted will be influenced by both the focus of the included articles (and any bias therein) and our framework of categorisation, counting was deemed the best way of obtaining an indication, albeit crude, of which tactics and arguments are relied upon most heavily by the TI.

The geography of where each tactic and argument was used was also identified. If the article included was transnational, wherever possible the geography of where the individual tactics and arguments were used was listed. For example, the article by ASH [36] is a transnational study but the tactic ‘indirect lobbying’ was used in the UK (Europe).

## Results

### Geography

In total 48 articles that mentioned arguments and tactics used by the TI when attempting to influence marketing regulation were included within this review. Over half (56%) of the articles focussed on activity in North America, Europe or Australasia, 17% focussed on Asia (Table 1). Only one article focussed on activity in Africa, but one ‘transnational’ study [37] also made references to TI arguments used when countering regulations proposed in Africa.

**Table 1:** Geographical location of TI activity.

Geographical Location	Number of articles (%)	Articles
Africa	**1** (2%)	**Africa** [38]
Asia	**8** (17%)	**Philippines** [39] **; Malaysia** [40] **; Japan** [41] **; Cambodia** [42] **; Lebanon** [43] **; China** [44] **; Uzbekistan** 1 [45] **; Middle East** [46]
Australasia	**3** (6%)	**Australia** [47] [48] **; New Zealand** [49]
Europe	**5** (10%)	**Switzerland** [50] **; Hungary** [51] **; Czech Republic** [52] **; European Union** [53] [54]
North America	**19** (40%)	**USA** [55] [56] [57] [58] [59] [60] [61] [62] [63] [64] [65] [66] [67] [68] [69] [70] [71] [72] [73]
South America	**4** (8%)	**Argentina** [74] **; Uruguay** [75] **; Latin America** [76] [77]
Transnational	**8** (17%)	**Transnational** [36] [78] [79] [80] [81] [37] [82] [83]
***Total***	***48***	

1 Different official bodies class Uzbekistan as either a Central Asian or European country. We have categorised it as Asian, as per the UN [84].

### Tactics and Arguments

The TI uses a number of recurring tactics (Table 2) and arguments (Table 3) when attempting to influence marketing regulation.

**Table 2:** Tactics used by the TI when attempting to influence marketing regulation.

Strategy (number of times identified)	Tactic		Number of times identified, by geography
Information (44)	Direct lobbying (meetings and correspondence with legislators/policymakers)		**23 : Africa –1** [38]; **Asia –3** [45] [43] [46]; **Australasia –2** [48] [37]; **Europe –7** [36] [36] [54] [53] [53] [51] [52]; **N.America –6** [65] [71] [63] [70] [37] [82]; **S.America –3** [77] [76] [74]; **Transnational –1** [81]
	Indirect lobbying (using third parties, including front groups, to lobby on the industry’s behalf)		**10 : Europe –5** [36] [36] [54] [53] [52]; **N.America –5** [58] [68] [71] [61] [37]
	Shaping the evidence base	Commissioning, writing (or ghost writing), or disseminating research/publications^1^	**7 : Australasia –2** [47] [48]; **Europe –2** [53] [51]; **N.America –3** [71] [36] [37]
		Preparing position papers, technical reports or data on impacts (including economic impact studies)	**2 : Europe –2** [54] [53]
	Establishing industry/policymaker collaboration (e.g. via working group, technical group, advisory group)/work alongside policymakers providing technical support/advice		**2 : Europe –2** [54] [51]
Constituency building (42)	External constituency building	Form alliances with and mobilise other industry sectors/business/trade organisations	**15 : Asia –1** [46]; **Australasia –1** [48]; **Europe –5** [36] [53] [53] [52] [51]; **N.America –6** [68] [60] [71] [63] [66] [37]; **S.America –2** [77] [76]
		Media advocacy (press releases, publicity campaigns, public hearings, interviews)	**7 : Europe –3** [50] [54] [53]; **N.America –4** [71] [37] [82] [83]
		Form alliances with or mobilize unions/civil society organizations/consumers/employees/the public	**6 : Australasia –1** [48]; **Europe –3** [54] [53] [53]; **N.America –2** [60] [61]
		Creation of front groups or astroturf organisations^2^	**3 : Europe –2** [53] [51]; **N.America –1** [60]
	Internal constituency building	Collaboration between companies/development of pan-industry group or industry trade association^3^	**11 : Asia –1** [40]; **Australasia –2** [47] [48]; **Europe –3** [36] [54] [51]; **N.America –2** [36] [37]; **S.America –2** [77] [76]; **Transnational –1** [81]
Policy substitution^4^ (32)	Develop/promote (new or existing) voluntary code/self-regulation		**18 : Asia –7** [40] [45] [41] [42] [43] [44] [46]; **Australasia –1** [47]; **Europe –5** [36] [50] [53] [52] [51]; **N.America –2** [55] [62]; **S.America –2** [77] [76]; **Transnational –1** [81]
	Develop/promote alternative regulatory policy^5^		**8 : Asia –1** [46]; **Australasia –1** [48]; **Europe –1** [53]; **N.America –2** [71] [64]; **S.America –2** [77] [74]; **Transnational –1** [83]
	Develop/promote non-regulatory initiative (generally seen to be ineffective/less effective, e.g. education programmes)		**6 : Africa –1** [38]; **Asia –1** [46]; **N.America –1** [83]; **S.America –2** [76] [74]; **Transnational –1** [81]
Legal (15)	Pre-emption		**6 : N.America –6** [55] [69] [70] [63] [64] [66]
	Using litigation/threat of legal action		**9 : Africa –2** [37] [37]; **Asia –1** [39]; **Australasia –1** [48]; **Europe –2** [54] [79]; **N.America –3** [57] [60] [72]
Constituency fragmentation and destabilization (2)	Preventing the emergence of, neutralising and/or discrediting potential opponents (individuals, organisations or coalitions)		**2 : N.America –2** [71] [61]
Financial Incentive (2)	Providing current or offering future employment to those in influential role		**1 : Europe –1** [53]
	Gifts, entertainment or other direct financial inducement		**1 : Europe –1** [52]

1 Including research/publications intended to undermine or misrepresent existing evidence.

2 Creation of group for specific purpose of working against proposed policy.

3 Routine use of a trade association was not counted, industry collaboration had to be ‘active’.

4 Includes efforts to prevent the implementation of ‘anticipated’ policies.

5 In some cases, industry uses legislators to promote their alternative policies.

**Table 3:** Arguments used by the TI when attempting to influence marketing regulation.

Frame (number of times identified)			Argument	Number of times identified, by geography
Negative Unintended Consequences (32)	Economic (21)	Manufacturers (10)	The cost of compliance for manufacturers will be high/the time required for implementation has been underestimated	**6 : Australasia –2** [48] [49]; **Europe –2** [54] [79]; **N.America –1** [65]; **Transnational –1** [78]
			Regulation will result in financial or job losses (among manufacturers)	**3 : Asia –1** [45]; **Europe –1** [54]; **N.America –1** [37]
			The regulation is discriminatory/regulation will not affect all producers/customers equally	**1 : Europe –1** [54]
		Public Revenue (7)	Regulation will cause economic/financial problems (for city, state, country or economic area (e.g. European Union))	**7 : Asia –2** [45] [46]; **Europe –2** [54] [51]; **N.America –3** [65] [67] [36]
		Associated industries (4)	Regulation will result in financial or job losses (among retailers and other associated industries, e.g. printing, advertising, leisure)	**4 : Australasia –1** [49]; **Europe –1** [54]; **N.America –2** [67] [66]
	Public Health (4)		Regulation will have negative public health consequences	**4 : Australasia –1** [48]; **N.America –2** [36] [37]; **Transnational –1** [80]
	Illicit Trade^1^ (2)		Regulation will cause an increase in illicit trade	**2 : N.America –2** [36] [37]
	Other (5)		Regulation could have other negative unintended consequences (e.g. cause confusion amongst customers, set a precedent for other types of products/’slippery slope’)	**5 : Africa –1** [37]; **Australasia –1** [49]; **N.America –2** [71] [36]; **Transnational –1** [78]
Legal (30)			Infringes legal rights of company (trademarks, intellectual property, constitutionally protected free speech (e.g. US First Amendment), international trade agreements)	**20 : Africa –2** [37] [37]; **Asia –3** [36] [37] [37]; **Australasia –3** [48] [37] [37]; **Europe –5** [36] [36] [54] [37] [37]; **N.America –4** [36] [56] [37] [37]; **S.America –1** [75]; **Transnational –2** [80] [37]
			Regulation is more extensive than necessary/regulation is disproportionate	**4 : Australasia –1** [48]; **Europe –1** [54]; **N.America –1** [37]; **Transnational –1** [80]
			Body doesn’t have the power to regulate/it’s beyond their jurisdiction	**4 : Europe –2** [54] [53]; **N.America –2** [57] [37]
			Regulation will cause an increase in compensation claims	**2 : Australasia –1** [37]; **N.America –1** [37]
Regulatory Redundancy (13)			Industry adheres to own self-regulation codes/self-regulation is working well	**5: Asia –1** [45]; **Australasia –1** [47]; **N.America –2** [59] [83]; **Transnational –1** [81]
			Industry only markets to those of legal age/is actively opposed to minors using product	**4 : Asia –1** [44]; **N.America –2** [58] [59]; **Transnational –1** [81]
			Existing regulation is satisfactory/existing regulation is satisfactory, but requires better enforcement	**4 : Europe –1** [54]; **N.America –3** [58] [59] [73]
Insufficient Evidence (11)			There’s insufficient evidence that the proposed policy will work/marketing doesn’t cause or change behaviour (it’s only used for brand selection and capturing market share), so regulation will have no effect	**10 : Asia –2** [45] [44]; **Australasia –4** [47] [48] [49] [78]; **Europe –1** [54]; **N.America –2** [36] [59] **; Transnational –1** [80]
			The health impacts of consumption remain unproven	**1 : Asia –1** [45]

1 ‘Illicit Trade’ is separate as it both undermines public health policy and has economic consequences.

#### TI tactics used to influence marketing regulation

This review identified 18 separate tactics (Table 2) falling under six main strategies: ‘Information’ (providing or manipulating evidence), ‘Constituency building’ (forming alliances with other sectors, organisations, or the public to give the impression of larger support for the industry’s position), ‘Policy substitution’ (proposing or supporting alternative policies), ‘Legal’ (using the legal system), ‘Constituency fragmentation, and destabilization’ (weakening opponents), and ‘Financial incentive’ (offering direct or indirect monetary incentives); further details included in Appendix S1.

The strategies ‘Constituency fragmentation’ and ‘Financial incentive’ were documented least frequently and each only in a single geographic region (North America and Europe respectively). The other strategies were widely used, although two individual tactics were only documented in single jurisdictions: pre-emption (Legal strategy) was only seen in the USA, although used in multiple states; and the preparation of position papers/technical reports (Information strategy) was only seen in the European Union. A further Information tactic, establishing collaboration with or working alongside policymakers, was only identified in Europe but within two jurisdictions.

A variety of Information strategies were identified and widely used. These include direct [36], [37], [38], [43], [45], [46], [48], [51], [52], [53], [54], [63], [65], [70], [71], [74], [76], [77], [81], [82] and indirect [36], [37], [52], [53], [54], [58], [61], [68], [71] lobbying of policymakers, attempting to shape the evidence via commissioning research [36], [37], [47], [48], [51], [53], [71] or preparing technical reports [53], [54], and efforts to establish collaboration with policymakers [51], [54]. When lobbying directly the TI often identifies and targets specific politicians, hoping that they will act on their behalf in policy discussions; this was seen in the UK [36], [53], Uzbekistan [45], Australia [48], the EU [54], and the USA where there was evidence that legislators have lobbied on the industry’s behalf [58], [68]. There was also evidence that the TI lobby domestic political actors to represent their interests in other countries; for example in 1992 when plain packaging was proposed in Australia, the TI “approached the vice-consul (commercial) of the British Consul General in Sydney” in order to ask for assistance from the UK government in dealing with the Australian government [37].

The use of indirect lobbying, where the TI’s interests are often hidden behind front groups or allies from other industry sectors or trade organisations, was also frequent. Evidence from only Europe and North America may reflect the loss of TI credibility in these regions and hence its need to use third parties, or it may simply reflect the research base. Examples illustrate the range of contexts in which this tactic was used. When opposing the UK’s Tobacco Advertising Bill in 2000, for example, the TI “encouraged a range of other organisations” to lobby the government on their behalf, these included the British Brands Group, the Association of Convenience Stores, and the Advertising Association [36], the TI again involved similar front groups when plain packaging was discussed in the UK in 2008 [36]. When the use of new health warnings was proposed in Australia in 1991, the TI, through a lobbyist, gained the support of third parties such as the Business Council of Australia, the Confederation of Australian Industry, the media, unions, advertising organisations, and growers and suppliers [48]. And when the European Community sought to end all tobacco advertising in member states in the 1990s, “PM [Philip Morris] sought to preserve Denmark’s opposition to the ban though the creation of the Committee for Freedom of Commercial Expression” which was “managed at arm’s length” and recruited more than 50 prominent Danes including a leading lawyer, a leading Danish writer and philosopher, and a well-known architect [53].

Attempts to shape the evidence base were also identified as a key element of the TI’s information strategy [36], [37], [47], [48], [51], [53], [54], [71]. *For example, in order to counter the EC directive banning tobacco advertising and sponsorship, the TI commissioned two “separate but complementary projects” through the Adam Smith Institute in London which were to argue against the ban in the “context of a host of proposals which progressively restrict personal freedom”* [*53*] *. W*hen plain packaging was suggested in Canada, the TI developed a common strategy and created the ‘Plain Packs Bible’ as a “resource for … the industry and allied groups who need to put the industry’s case in public” and something that would be “accessible for civil servants and politicians” [36].

External Constituency Building was often linked to indirect lobbying because the TI both creates front groups or astroturf organisations to lobby on its behalf [51], [53], [60], or forms alliances with and mobilises existing organisations [36], [37], [46], [48], [51], [52], [53], [54], [60], [61], [63], [66], [68], [71], [76], [77]. For example, PM was able to cultivate and create allies to support the TI by contributing financially to women’s organisations in the USA; when a bill further restricting television advertising was proposed, American Women in Radio and Television wrote letters to Congress opposing the ban “out of gratitude” for PM’s support [61].

Internal Constituency Building (collaboration among manufacturers) was also common and cut across different policies and jurisdictions [36], [37], [40], [47], [48], [51], [54], [76], [77], [81]. This review suggests it occurs when the TI is facing a major regulatory threat, for example it was reported in TI attempts to combat the introduction of plain packaging in Canada [36], the FCTC globally [81], and the European Tobacco Products Directive (TPD) [54].

The use of policy substitution also appears to be a key strategy used to prevent the implementation of marketing regulations, and has been documented globally. Most frequently the TI proposes the implementation of voluntary regulation in place of formal legislation [36], [40], [41], [42], [43], [44], [45], [46], [47], [50], [51], [52], [53], [55], [62], [76], [77], [81]. This tactic is designed to reduce political pressure to formally regulate (which is attractive to policymakers, mostly due to cost), and to pre-empt political action. For example, when faced with a ban on all direct and indirect advertising in Malaysia, the TI created a voluntary code entitled ‘Code for the Marketing of Cigarettes’ [40], and similarly in Australia Philip Morris International (PMI) developed their own marketing code of practice specifically intended to be used “in lobbying, to gain a public relations advantage by promoting PMI as responsible towards youth” [47]. Voluntary regulation has also been proposed by the TI, and in some cases implemented, in the USA [55], [62], Japan [41], Cambodia [42], Lebanon [43], China [44], Hungary [51], Czech Republic [52], UAE [46], and Switzerland [50], and has also been suggested when the risk of regulatory intervention is transnational [53], [76], [77], [81].

Similarly, the TI often develops or promotes non-regulatory initiatives, such as youth education programmes, in order to avoid more formal legislation and appear socially responsible [38], [46], [74], [76], [81], [83]. For example, when opposing the FCTC restrictions on marketing, the TI saw youth access schemes as a way to “make a significant gesture that would divert attention from the FCTC, moderate the WHO’s moves toward the FCTC, and bring the tobacco companies together against the FCTC” [81]. Much research shows TI-funded educational campaigns are ineffective [83], [85], and often counterproductive [85], [86].

The third policy substitution tactic identified is the development or promotion of an alternative regulatory policy to the one being proposed that is less effective and more favourable to their business interests [46], [48], [53], [64], [71], [74], [77], [83]. For example, in Australia, when new health warnings were proposed in 1991, the TI decided that their best chance of minimising their effect was to support the adoption of the (weaker) European health warnings then being used, rather than those suggested by the Australian Ministerial Council on Drugs Strategy, as the European warnings were at the bottom of the pack and comparatively small [48]. And, when the European Community proposed a ban on tobacco advertising, the TI in Germany worked with the German government to “introduce a weak proposal designed to replace the proposed, stronger EC advertising ban” [53]. It was drafted by TI officials and was meant to be submitted to the EC through German representatives without acknowledging its true origin [53].

Using or threatening legal action against a proposed regulation was commonly used and seen globally [37], [39], [48], [54], [57], [60], [72], [79], for example it was used multiple times when packaging regulations were proposed [37], [39], [72], [79]. It is typically used once other tactics have failed, and reinforces industry arguments about the high costs of regulation and the immediate fiscal advantage of policies (notably self-regulation) promoted by the industry. The TI was also seen attempting to use legal action to suppress an individual opposition organisation [48]. The second legal tactic, pre-emption, was only documented in the US but occurred in multiple states. It was found to have been used when the TI was arguing against youth access restrictions [55], [63], [64], [70], and in some cases more specifically against vending machines restrictions [66], [69].

#### TI arguments used to influence marketing regulation

This review identified 17 separate arguments (Table 3), which were grouped into four main frames: ‘Negative Unintended Consequences’ (direct and indirect compliance costs (monetary and other)), ‘Legal’ (illegality of the policy (the implicit cost for government)), ‘Regulatory Redundancy’ (policy is unnecessary), and ‘Insufficient Evidence’ (policy is not based on sound evidence); further details included in Appendix S1. The ‘Negative Unintended Consequences’ and ‘Legal’ frames were the most commonly used.

While all of the frames were seen across a wide range of geographic areas, again highlighting the cross-national nature of TI political activity, three arguments within these frames were only used in one jurisdiction: “Regulation will cause an increase in illicit trade” was only identified as having been used in the USA [36], [37]; the argument that ‘the regulation is discriminatory’ was only identified as having been used once in Europe in relation to the TPD [54]; and the argument that ‘the health impacts of smoking remain unproven’ was only identified in Uzbekistan [45].

A large number of arguments focused on the negative unintended consequences of legislation. These included claims of economic losses to tobacco manufacturers (both compliance costs and job losses) [37], [45], [48], [49], [54], [65], [78], [79], associated industries [49], [54], [66], [67], and the public revenue [36], [45], [46], [51], [54], [65], [67]. Such arguments sometimes involve exaggerated claims, for example it was claimed that a ban on advertising in Uzbekistan would lead to “the immediate demise of the domestic cigarette industry” [45]. There are a wide variety of other arguments in this frame, including the claim that the proposed regulation will have negative public health consequences [36], [37], [48], [80]. For example the TI argued that plain packaging in Canada would “make cigarettes cheaper and more available to youth” [37] and new warning labels in Australia would lead to “warning overload” causing consumers to “ignore warning labels entirely” [48].

Arguments questioning the legality of policies to curb TI marketing (legal frame) are very common and aim to shift the focus of the debate away from public health and consumer protection, aiming instead to highlight the potential administrative costs of new policies. The argument that a proposed policy contravenes the TI’s legal rights has been widely used to fight a variety of public health policies including the regulation of packaging (health warnings and plain packaging) [36], [37], [48], [75], [80], product descriptors (such as ‘light’ or ‘mild’) [36], [37], and advertising bans [37], [56]. Such arguments frequently claim that public health measures are incompatible with trade law. For example FCTC proposals to remove product descriptors were met with TI arguments that the words were part of a trademark and therefore the proposed regulation would violate TRIPS and the Paris Convention [37] and in Uruguay PM alleged that plain packaging regulations would violate a Switzerland-Uruguay bilateral investment treaty [75]. Such arguments are made despite growing evidence that they are misplaced (see below).

Other arguments falling within the legal frame involve exaggerated and emotive claims such that regulation is “extreme”, “disproportionate” (PM on plain packaging [80]), or “excessive” (the TI on pack health warnings in Canada [37]), or a manifestation of “nanny state” tendencies (as per Australia’s approach to health warnings [48]). These claims relate to broader libertarian arguments about the appropriate level of state intervention and regulation. In Europe, the TI has argued that the EU’s powers do not extend to regulating on public health, and the issue is instead one for individual Member States [53], [54], and similarly in North America the TI has argued that regulation is “beyond federal jurisdiction” [37] and that “the Constitution prohibits any one state from regulating avenues of national commerce” [57]. Although the argument that the proposed regulation will lead to an increase in compensation claims was only identified twice [37], the threat of the cost of litigation underlies all of these legal arguments.

Arguments that the regulation is unnecessary (regulatory redundancy frame) were also frequently used. These claims took a variety of forms including that the TI is opposed to youth smoking and does not market to youth [44], [58], [59], [81], and that they can be trusted to comply with voluntary regulations and that existing voluntary initiatives work [45], [47], [59], [81], [83]. In some instances the TI suggested that existing regulation was sufficient or simply needed better enforcement [54], [58], [59], [73]. In all cases the overarching message was that further regulation was unnecessary.

Questioning the strength of evidence [36], [44], [45], [47], [48], [49], [54], [59], [78], [80] (insufficient evidence frame) favourable to public health policies is a common technique that has been used, for example, to oppose the introduction of plain packaging (in New Zealand [78], Canada [36], and transnationally [80]) and raise doubts about the impact of TI advertising on consumer behaviour [45], [47], [59]. This argument, along with others, is used to increase scepticism about the likely benefits of regulation and reinforce other arguments the TI makes.

There are major doubts over the accuracy of almost all of the arguments identified in this taxonomy. For example, asserting that plain packaging violates trademarks under World Trade Organisation (WTO) rules ignores the distinction made between registration and use under TRIPS and the Paris Convention [87]. There is no provision within WTO rules that requires “WTO Members to grant the owner of a registered trademark, an affirmative right to actually ‘use’ that mark” [87]. Moreover, members are granted “significant flexibility in enacting public health measures” when it’s necessary to protect “human, animal or plant life or health” [87]. While the US First Amendment provides extensive protection to freedom of speech, it has been argued that “protecting the public health may necessitate stringent limits on commercial expression” [88] therefore allowing some speech to be restricted for the good of the public’s health. In Europe and North America where the TI have argued that the regulation falls outside of EU or federal jurisdiction is also false, and its attempts to overturn regulation on this basis have failed [54]. Additionally, arguing that there is no evidence that regulating marketing works has also been found to be false. Much research has found a significant link between advertising and smoking behaviour [9], [10], [11], [12], [13], [14], [15], including evidence of a relationship between exposure to smoking in films and adolescent smoking [89], [90], [91].

## Discussion

This systematic review suggests that the TI uses a relatively narrow range of strategies/tactics and frames/arguments when attempting to influence marketing regulation, albeit a wider range than suggested by existing taxonomies of corporate political activity. This review also suggests that TI political activity is not geographically specific, with strategies/tactics and frames/arguments being used across a wide variety of jurisdictions. Consequently the taxonomies developed within this paper are likely to be helpful in understanding TI political activity internationally.

### Taxonomies

Hillman and Hitt’s [21] framework, on which the categorisation of tactics in this review was initially based, considerably under-represents the range of tactics that the TI uses when attempting to influence policy. This may reflect both the unprecedented number of regulatory risks facing this particular industry and that their categorisation was developed prior to the release of internal TI documents. Furthermore Hillman and Hitt’s [21] taxonomy was based on exchange theory which assumes that corporate political activity represents one side of an exchange relationship in which corporations offer policymakers support and information in return for influencing policy. While the relevance of this approach is now arguably more limited with the advent of the FCTC’s Article 5.3 (which aims to protect public health policies from the “vested interests of the tobacco industry” [92]), this will not necessarily reduce the TI’s ability to influence policy but simply require them to do so less directly. The frequency with which the TI relies on third parties highlights the weakness of exchange theory-based models of corporate political activity. We also identified tactics/strategies that sit outside of exchange theory (such as constituency fragmentation, the threat of litigation, and ineffective forms of self-regulation) which challenges the assumption that corporate political activity is designed to produce outcomes that are mutually beneficial to corporations and policymakers, and we show that the information and arguments the TI uses are highly misleading; findings which suggest the original model may be both limited and naïve.

Although it appears that the TI uses a number of discrete arguments within a narrow range of frames, many of them fall within a larger ‘cost-benefit’ meta-frame which promotes the economic and social costs of proposed public health policies and underplays their benefits. This approach is highly relevant to current policymaking which embeds stakeholder consultation and impact assessments within the process of policy formation. It has previously been shown that the TI successfully lobbied for the introduction of impact assessments in Europe (impact assessments using a cost-benefit approach in which the impacts of policies are monetised) because it felt that this system would work to its advantage and make it harder for public health policies to be implemented [93]. This is also supported by the related literature [94] which shows how impact assessment, notably cost benefit analysis, can serve to assist corporate interests. Arguments such as ‘the cost of compliance will be high’, ‘the regulation is more extensive than necessary’ and those under the ‘negative unintended consequences’ frame are used to increase scepticism about the likely benefits of regulation, and highlight the potential future costs for industry, retailers, and the public through the wasting of public funds on unnecessary policy formation, discussion and implementation. This is also observed through the omission of a ‘health’ frame [95]; this review found no evidence of the TI making reference to the dangers of smoking, although it did find an example of the TI refuting the relationship between smoking and disease as late as 1994 in Uzbekistan [45].

Finally we note that there is some overlap in the tactics and arguments used by the TI. For example, there is both a legal strategy and a legal frame, the policy substitution strategy overlaps with the regulatory redundancy frame (especially, for example, the tactic ‘develop/promote voluntary code/self-regulation’ and argument ‘industry adheres to own self-regulation’), and many of the arguments within the negative unintended consequences frame are linked to efforts in constituency building. This highlights how the tactics and arguments used by the TI are mutually reinforcing.

### Strengths and Limitations

This review has a number of limitations. First, although a broad search strategy and search string was used when initially identifying articles it is still possible that some relevant articles may have been missed and therefore not included within the review. To minimise this, we worked with a librarian, searched online research repositories, and contacted experts in the field to identify additional articles. Second, the coding of arguments and tactics within the articles is often subjective. To mitigate this, all three authors reviewed and re-reviewed the coding at various points during the systematic review process and, at the end, collectively reviewed 50% of the included articles, plus all of those in categories where coding concerns had been identified. Third, the identification of tactics and arguments, and the jurisdictions in which they are used, is dependent on the available literature, its quality, and any publication bias. This in turn may depend on limitations in the availability and nature of the TI documents on which much of the literature is based. These issues have a number of implications. For example, many of the articles included did not focus primarily on TI attempts to influence marketing regulations and thus only made brief references to TI tactics or arguments, with little context or background. We attempted to overcome this limitation by requiring each tactic and argument to be supported by verifiable evidence. Information regarding the success or failure of a particular policy proposal was not always recoded, making it impossible to reliably determine which tactics or arguments were most successful in defeating marketing-related regulations. Furthermore, it is highly likely that some of the tactics and arguments were used more frequently by the TI than identified within the literature. For example, financial incentives are likely to be used more frequently and broadly than the two occasions identified in Europe [52], [53], we know that the TI frequently attempts to discredit their opponents (see for example [96], [97], [98], [99]), however this tactic was only found to have been used twice within the included literature [61], [71], and similarly, arguments that marketing regulations will increase illicit tobacco are more commonly used, and in more jurisdictions, than this review would suggest [100], [101], [102]. The limited appearance of some arguments, such as tobacco not having been proven to cause disease (which was only identified as having been used by the TI in Uzbekistan in 1994 [45]), may reflect the fact that we only examined tactics and arguments from 1990 onwards. In addition, we note that despite a growing literature showing how the TI influences trade agreements and then uses them to argue against the feasibility of regulations [103], [104], the use of trade agreements to pre-empt marketing policy is not identified as a tactic (although we do identify the use of trade agreements as an argument under the ‘legal’ frame). This is perhaps due to the focus of our search being on the TI’s influence of marketing regulations which may, therefore, have missed articles examining industry influence on trade agreements that were in turn used to influence marketing regulations. Due to our concerns regarding bias in the literature, the counting element of this review should be used as a guideline only to provide some insight into the most frequently used tactics and arguments.

The main strength of this review is its systematic approach and its attempt to rigorously categorise industry strategies/tactics and frames/arguments; to our knowledge it is the first attempt to do so. A key strength is the geographic diversity of the literature reviewed. Although over half of the included articles (26 articles, 56%) focussed on North America, Europe or Australasia (perhaps in large part due to grants provided by the US National Cancer Institute for research on TI documents in the early 2000s), a significant proportion did not, and the geographic base was far more diverse than some previous reviews of industry tactics [1]. While some tactics and arguments were seen only in one or a few jurisdictions, this sometimes appears to reflect limitations in the underlying literature (see above), or specific jurisdictional issues for example the use of pre-emption in the USA. While care needs to be taken in assuming that tactics and arguments used in one jurisdiction will be used elsewhere, this review suggests that the findings will be broadly applicable across different geographies. It is, however, also important to recognise that some arguments are likely to be more effective in certain circumstances, for example legal arguments may be more successful where government legal expertise is undeveloped and the costs of litigation proportional to government revenue are high [103].

### Implications for Policy, Practice, and Research

This systematic review has identified common tactics and arguments that the TI uses to prevent the implementation of regulation, and has shown that they are repeatedly used across different jurisdictions. Policymakers need to be aware of these in order to understand how the TI may try to manipulate the regulatory environment in their own interests, and public health advocates can use this information to prepare effective counter strategies. The recent failure of the British government to pursue plain packaging legislation highlights the importance of such knowledge.

Models of corporate political activity based on internal TI documents represent a potentially valuable analytical tool with which to examine opposition to public health policies and identify low visibility activity. The repeated use of tactics and arguments identified in this review underlines the potential of such an approach. Further work is now needed to examine whether the taxonomies for TI tactics and arguments developed in this paper can be applied to other industries and policy areas. Further research is also required to examine the interconnections between strategies/tactics, frames/arguments and audiences, as different social actors are likely to propagate different messages and have different political effects. Finally, due to limitations in the included literature, we also recommend that future research on corporate influence should, where at all possible, include contextual information, ensure all claims are supported by reliable and verifiable evidence, and that the success or failure of individual tactics and arguments are recorded.

## Supporting Information

Appendix S1 (PDF)

Checklist S1 PRISMA Checklist for this study.
(PDF)

Flow Diagram S1 (PDF)

Protocol S1 (PDF)

Box S1 (PDF)
