# Supplementary material for: How Does the Tobacco Industry Attempt to Influence Marketing Regulations? A Systematic Review
Source: PLoS One. 2014 Feb 5;9(2):e87389. doi: 10.1371/journal.pone.0087389 (PMC3914831; doi:10.1371/journal.pone.0087389)
Supplement: Appendix S1 — (PDF) [file pone.0087389.s001.pdf]

Appendix

Taxonomies 2

Table 1: Description of Strategies and Frames 2

Table 2: Full list of industry strategies/tactics 5

Table 3: Full list of industry frames/arguments 7

Data Extraction 9

Table 4: Summary of studies relating to tobacco industry (TI) attempts to influence marketing-related regulation. Only the tactics and arguments recorded/counted are listed. 9

Explanation of systematic searches 81

Table 5: Searches completed and the number of articles returned 81

Taxonomies

Table 1: Description of Strategies and Frames

Category	Description
**Strategies**	
**Information**	Policymakers face considerable problems in producing information which can accurately predict the economic and health consequences of policies, whilst businesses typically have access to policy-relevant information, relating, for example, to employment figures or to the nature of product ingredients and their health impacts. Businesses take advantage of these information asymmetries by supplying policymakers with information about the likelihood of adverse effects of a pending policy. Exchange theory assumes that information supplied by business represents a form of subsidy, which invariably aids optimal policy outcomes. In practice, however, policymakers often lack the information necessary to assess the accuracy or veracity of industry predictions. As the tactics summarised in this systematic review illustrate, tobacco companies exploit this information inequity by providing policymakers with false or misleading information by understating the health benefits of a proposed policy and overstating its social and economic consequences.
**Constituency building**	Constituency Building involves gaining the support of other sectors, organisations, or individuals in order to give the impression of a larger support-base for the industry position. Because the outcome of policy discussions in contemporary democracies is often determined by the success or failure of efforts to enlarge their scope, constituency building is a key political strategy of both public health and business actors. Both elected and non-elected officials attach a premium to constituent support. Industry approaches constituency building in three key ways: forming alliances with other organisations (such as trade associations, unions, or organisations representing other industry sectors); mobilising the public (through publicity campaigns, increasingly through digital media, and press releases); and via false alliances built through the creation of front groups and astroturf organisations[1].
**Financial incentive**	Policymakers respond to financial inducements by business actors. The general literature focuses on campaign finance and other political donations, whereas tobacco industry document-based studies also highlight ‘under-the-counter’

	financial inducements such as making offers of employment, gift giving, and the provision of entertainment.
**Policy substitution**	In addition to financial inducements, offers of support (Constituency Building), and information subsidies, business actors can also provide policy subsidies, where alternative policies are developed as a substitute for proposed policies. Policy substitution is attractive to policymakers as it reduces the administrative costs associated with developing and implementing policies, whilst still appearing to meet objectives. Voluntary codes represent the most common form of policy substitution; although document-based studies also highlight the tobacco industry's development of alternative regulatory policies. In practice, both voluntary and regulatory industry alternatives are usually less effective at promoting public health.
**Legal**	Using or threatening legal action against proposed policies is often used when other, less adversarial political tactics have failed. Litigation (and threats of litigation) work by raising the (perceived and actual) costs associated with implementing regulation, and underline the immediate fiscal advantages of voluntary codes and regulatory policies acceptable to the industry.
**Constituency fragmentation and destabilization**	As well as building supportive constituencies (Constituency Building), the industry also works to weaken opposing constituencies or prevent them from emerging in the first place. Amongst other things, document-based studies note tobacco industry efforts to discredit opponents and manufacture divisions within opposing political consistencies supportive of public health policies. The aim is to intimidate opponents, hoping to stop or reduce opposition to industry actions, or make opponents appear extreme or disreputable.
**Frames**	
**Negative Unintended Consequences**	The industry uses a wide range of arguments to highlight the potential negative effects of regulation. These 'negative unintended consequences' include economic impacts (such as financial or job losses for manufacturers, losses to the public revenue, and losses to associated industries such as the hospitality and advertising industries), public health impacts (where the risk of regulation causing harm to health through, for example, increased consumption, is highlighted), and a wide range of other impacts, including setting a precedent for other types of products and causing confusion amongst customers. The argument that proposed regulations will increase illicit trade

	feeds into many of the other 'negative consequence' arguments, as increases in illicit trade can have both economic and public health impacts (by making the product cheaper and more available).
**Legal**	Legal arguments highlight the illegality of proposed policies. They generally aim to shift the focus of the debate away from public health and consumer protection, and instead highlight the potential administrative costs of new policies.
**Regulatory Redundancy**	The industry uses a number of arguments which seek to emphasise that proposed policies are unnecessary, and liable to waste public funds. These points are conveyed through, for example, industry claims that it is capable of self-regulation, and that existing regulation is satisfactory (and simply in need of better enforcement).
**Insufficient Evidence**	The industry uses a number of arguments aimed at questioning the strength of the evidence base behind proposed health policies. Such arguments aim to increase scepticism of the likely benefits of proposed policies and support arguments from other frames, which emphasise the costs and underplay the benefits of public health policies.

Table 2: Full list of industry strategies/tactics

Strategy	Tactic	
Information	Direct lobbying (meetings and correspondence with legislators/policymakers)	
	Indirect lobbying (using third parties, including front groups, to lobby on the industry’s behalf)	
	Shaping the evidence base	Commissioning, writing (or ghost writing), or disseminating research/publications[1]
		Preparing position papers, technical reports or data on impacts (including economic impact studies)
	Establishing industry/policymaker collaboration (e.g. via working group, technical group, advisory group) / work alongside policymakers providing technical support/advice	
Constituency building	External constituency building	Form alliances with and mobilise other industry sectors/ business/ trade organisations
		Media advocacy (press releases, publicity campaigns, public hearings, interviews)
		Form alliances with or mobilize unions/civil society organizations/ consumers/employees/the public
		Creation of front groups or astroturf organisations[2]
	Internal constituency building	Collaboration between companies / development of pan-industry group or industry trade association[3]
Policy substitution[4]	Develop/promote (new or existing) voluntary code / self-regulation	
	Develop/promote alternative regulatory policy[5]	
	Develop/promote non-regulatory initiative (generally seen to be	

	ineffective/less effective, e.g. education programmes)
Legal	Pre-emption
	Using litigation / threat of legal action
Constituency fragmentation and destabilization	Preventing the emergence of, neutralising and/or discrediting potential opponents (individuals, organisations or coalitions)
Financial Incentive	Providing current or offering future employment to those in influential role
	Gifts, entertainment or other direct financial inducement

1. Including research/publications intended to undermine or misrepresent existing evidence.
2. Creation of group for specific purpose of working against proposed policy.
3. Routine use of a trade association was not counted, industry collaboration had to be 'active'.
4. Includes efforts to prevent the implementation of 'anticipated' policies.
5. In some cases, industry uses legislators to promote their alternative policies. For example, in McDaniel and Malone (2005) company representatives worked with legislators, meeting with staff to explain PM's views, helping to write legislation and lobbying on behalf of legislation PM supported.

Table 3: Full list of industry frames/arguments

Frame			Argument
Negative Unintended Consequences	Economic	Manufacturers	The cost of compliance for manufacturers will be high / the time required for implementation has been underestimated
			Regulation will result in financial or job losses (among manufacturers)
			The regulation is discriminatory / regulation will not affect all producers/customers equally
		Public Revenue	Regulation will cause economic/financial problems (for city, state, country or economic area (e.g. European Union))
		Associated industries	Regulation will result in financial or job losses (among retailers and other associated industries, e.g. printing, advertising, leisure)
	Public Health		Regulation will have negative public health consequences
	Illicit Trade[1]		Regulation will cause an increase in illicit trade
	Other		Regulation could have other negative unintended consequences (e.g. cause confusion amongst customers, set a precedent for other types of products/'slippery slope')
Legal			Infringes legal rights of company (trademarks, intellectual property, constitutionally protected free speech (e.g. US First Amendment), international trade agreements)
			Regulation is more extensive than necessary / regulation is disproportionate
			Body doesn't have the power to regulate / it's beyond their jurisdiction
			Regulation will cause an increase in compensation claims

Regulatory Redundancy	Industry adheres to own self-regulation codes / self-regulation is working well
	Industry only markets to those of legal age / is actively opposed to minors using product
	Existing regulation is satisfactory/existing regulation is satisfactory, but requires better enforcement
Insufficient Evidence	There's insufficient evidence that the proposed policy will work / marketing doesn't cause or change behaviour (it's only used for brand selection and capturing market share), so regulation will have no effect
	The health impacts of consumption remain unproven

1. 'Illicit Trade' is separate as it both undermines public health policy and has economic consequences.

Data Extraction

Table 4: Summary of studies relating to tobacco industry (TI) attempts to influence marketing-related regulation. Only the tactics and arguments recorded/counted are listed.

Study	Geography	What policy is the TI attempting to influence?	Tactics used	Arguments used
Alechnowicz and Chapman, 2004 [2]	The Philippines	In 1991, proposals for national legislation to introduce health warnings [on packs] resurfaced.	In 1994, the Philippines Industry Association commenced a lawsuit against regulation requiring that the side panel health warning be replaced by back and front notices covering 25% of both panels.	
Apollonio and Malone, 2010 [3]	USA	General youth access regulations	The tobacco industry and retailers anticipated from the program's inception that We Card could be used to block stronger policies restricting youth access to tobacco. Industry surveys in 1996 found that retailers saw this as an excellent use of the program…. Similarly, Tobacco Institute lobbyists viewed the program as primarily political, noting in a 1997 report: ''Once again, work by the WE CARD Coalition has been instrumental in state efforts to enact reasonable youth access	

Study	Geography	What policy is the TI attempting to influence?	Tactics used	Arguments used
			laws.'' As an example of ''reasonable'' laws, the memo praised state laws preempting stronger local legislation.	
ASH, 2010 [4]<br><br>(overlaps with [5] and [6])	Transnational	1. UK: The Health Act (2008/9)<br>2. Canada: Plain packaging (1993)<br>3. Hong Kong: Plain packaging (1995)<br>4. European Union: Ban on 'light' and 'mild' (2000)<br>5. UK: Tessa Jowell's Private Members Bill and the Lewis Bill (1994)<br>6. UK:	**1.**<br>When Alan Johnson, former secretary of state for health, launched the consultation on the future of tobacco control in Britain in may 2008, the tobacco companies, Philip Morris, British American Tobacco, Imperial Tobacco and Japan Tobacco International responded to the consultation paper to argue the case against the introduction of plain packaging….Front groups also made the same arguments: The European Communities Trade Mark Association (ECTA), The International Trademark Association (INTA), British Brands Group, The Anti Counterfeiting Group, Business Action to Stop Counterfeiting and Piracy (BASCAP), Trans Atlantic Business Dialogue (TABD), Global Intellectual Property Centre, Tobacco Control Accountability Initiative, Markenverband, Nude Brand Creation.<br><br>Imperial Tobacco wrote to the Department of Health and members of the house of lords giving notice that it would seek judicial review	**1.**<br>Imperial Tobacco wrote to the Department of Health and members of the house of lords giving notice that it would seek judicial review of any legislation barring branded packs. The letter stated, "Imperial Tobacco is also concerned about the continued erosion and potential expropriation of our valuable intellectual property rights… Regulation that requires plain packaging will expropriate valuable corporate assets in which the company and its shareholders have invested for more than a century and risks placing the UK government in breach of a range of legal and treaty obligations that relate to intellectual property rights, international trade and EU law." ... Japan Tobacco international (JTI) responded saying that plain packaging would be "in breach" of

Study	Geography	What policy is the TI attempting to influence?	Tactics used	Arguments used
		Tobacco Advertising Bill (2000)	of any legislation barring branded packs.<br><br>**2.**<br>The tobacco companies acted together. In 1993 a "Plain Pack Group" was formed representing British American Tobacco, RJR Tobacco International, Gallaher, Reemtsma, Rothmans, Benson & Hedges, Imperial, Rothmans International Services and Philip Morris International.<br><br>The tobacco companies came together in 1993 to counter the threat of plain packaging in Canada and developed a common strategy. They decided to create a 'plain packs bible'. John Luik was commissioned by the Plain Pack Group to do this.<br><br>Other "front men" recruited by the Plain Pack Group included John Murphy and Iain Mills. Mills was a Conservative Party politician in the United Kingdom, and in 1994 Mills advised Rothmans on how to influence parliamentarians against packaging reform. This was during a period when a Private members Bill had been tabled by Kevin Barron MP to ban tobacco advertising.	TRIPs and the Paris Convention.... BAT responded saying that Intellectual property rights are 'a cornerstone of economic activity', hence both their significant value to their owners and the wider economy and the need for them to be protected effectively at both the domestic and international levels. The UK Government is not entitled to interfere with trade mark and related intellectual property rights in respect of lawful products by reference to the nature of those products, because such an interference would be contrary to the harmonised EU and international system of trade mark protection with which it is obliged to comply." ... Philip Morris responded saying that "plain packaging will squarely conflict with" TRIPS and the Paris Convention.<br><br>**2.**<br>Together tobacco companies developed key messages including:

Study	Geography	What policy is the TI attempting to influence?	Tactics used	Arguments used
			**5.**<br>The Lewis-Jowell Working Group was convened, with all the major tobacco companies taking their part in setting out a plan of action. It was decided that the TMA would write to all potential supporters, while the companies would contact those MPs with whom they already had a relationship. They listed MPs they planned to target for support and advice from both sides of the house but notably John Carlisle, who later became the TMA's Public Affairs Director, and Iain Mills who offered them advice around the time of the Barron Bill.<br><br>**6.**<br>The tobacco industry also encouraged a range of other organisations including the British Brands Group, Association of Convenience stores and the Advertising Association to lobby the Government on the economic impact of an advertising ban…. seeing the writing on the wall in 2001, the TMA advised its members to make a last ditch attempt to reinstate the voluntary agreement.	• "Plain packaging will do nothing to achieve its [the Canadian Government's] anti smoking objectives at considerable potential cost in jobs, international trade problems and smuggling"<br>• "Plain packaging may be illegal"<br>• Plain packaging is "merely harassment of smokers"<br>• There was no evidence that plain packaging would reduce smoking and " could, in fact, have the opposite effect of that intended"<br>• "The assumption is that changes in the packaging will lead to changes in behaviour. There is no clear evidence that removing the trade mark, or dispensing with the pack design, will affect behaviour."<br>• "Prohibiting the use of trademarks is contrary to GATT"<br>• "Banning the use of any registered logo, design, pattern

Study	Geography	What policy is the TI attempting to influence?	Tactics used	Arguments used
				or trade mark amounts to censorship”<br>• “ . . .a trademark to be registered and protected, must be used. If use is prohibited, the protection afforded by registration may not be available.”<br>• Compromising tobacco trademarks would interfere with an ‘orderly tobacco market’, result in increased counterfeiting and smuggling and have significant economic consequences<br>• if adopted this would be an approach that might eventually be applied to alcohol and other products<br><br>**3.**<br>In 1995 the Tobacco Institute of Hong Kong told the Hong Kong government that its proposed smoking (Public health) (Amendment) Bill would diminish commercial value of trademarks and

Study	Geography	What policy is the TI attempting to influence?	Tactics used	Arguments used
				may violate the Paris Convention, GATT and TRIPS.<br><br>**4.**<br>In 2000 The Confederation of European Community Cigarette Manufacturers ltd briefed the EU in response to the proposed ban on light and mild descriptors and claimed it was a violation of TRIPS and the Paris Convention in the case of names like "Mild Seven".
Assunta and Chapman, 2004 [7]	Malaysia	Government efforts to regulate tobacco promotion and health warnings	One strategy proposed the adoption of codes of practice for cigarette marketing, TMD advertising, sponsorship, and sampling. In December 1992 the members of CMTM, namely Rothmans of Pall Mall (M), Malaysian Tobacco Company, RJ Reynolds (M), and Godfrey Phillips (M) signed an agreement among themselves to adopt a ''Code for the Marketing of Cigarettes''.	
Bond et al, 2009 [8]	USA	General marketing regulation		As part of PM's defence of advertising, a recurring theme is reference to the US First Amendment for protection against advertising restrictions on both alcohol and tobacco products.

Study	Geography	What policy is the TI attempting to influence?	Tactics used	Arguments used
				“Truthful advertising about legal products is protected by the First Amendment”.
Carter, 2003 [9]	Australia	General youth restrictions	In 1991 the Philip Morris group began ongoing work on a PMI marketing code of practice. This involved PMI’s presidents and vice presidents, senior counsel, regional presidents, field marketing staff, and senior management at PM Corporation. The code was intended to be used, particularly in lobbying, to gain a public relations advantage by promoting PMI as responsible towards youth, and to simultaneously protect PMI’s ability to be ‘‘competitive and creative’’ in its marketing.<br><br>The public version of PMI’s youth policy was instead encapsulated in a brochure, entitled ‘‘A global commitment to responsible marketing’’, for ‘‘legislators, journalists and decision makers from around the world’’.<br><br>In 1998 PMI went into negotiations with RJ Reynolds Tobacco International and BAT, after which they were ‘‘hopeful that we will be able to implement a greatly expanded joint international industry youth access and	The [public version of PMI’s youth policy] brochure intended to show that advertising did not cause youth smoking, and that PMI, with their marketing code, were the industry leaders on the issue and did not need to be regulated further.

Study	Geography	What policy is the TI attempting to influence?	Tactics used	Arguments used
			marketing code to which all major industry participants will voluntarily adhere''.	
Chapman and Carter, 2003 [10]	Australia	In 1991, the Ministerial Council on Drug Strategy (MCDS) directed its Tobacco Issues Committee to examine the case for introducing new health warnings.	PM was ''appointing a top man and devoting considerable resources to the matter'' and asked if (BAT's) Wills would ''upgrade their effort and work with us to create an industry platform''.<br><br>In 1992, the industry decided that their main chance was to promote the adoption of the proposed European Economic Community (EEC) health warnings. These warnings were at the bottom of the pack, comparatively small, and non-explicit, and under EEC agreements, nations would be able to select from between two and six out of 15 optional warnings. Heavily influenced by local tobacco industry lobbying, the three most popular warnings selected by the European nations were ''Smoking when pregnant harms your baby'' (nine nations adopting); ''Protect children: don't make them breathe your smoke'' (six nations); and ''Don't smoke if you want to stay healthy'' (six nations). Significantly, none chose ''Smoking causes addiction.''<br><br>The industry believed it could use its influence	PM's Henry Goldberg wrote to Jeff Kennett [Victorian Liberal opposition leader] noting that the MCDS recommendations were contrary to the position Kennett had stated publicly would be Victoria's position (that is, adopting the EEC warnings). Goldberg argued that ''proposals to radically alter packaging will do nothing to increase this awareness [of the health risks of smoking] and will do nothing to address concerns about smoking incidence in young people'' as well as appealing to Kennett's strongly conservative political agenda by framing warnings as an unfair burden on business.<br><br>When PM's Bill Murray attempted to persuade the NSW Premier on the industry's position in 1992, he argued that it was only ''…'nanny' states as we refer to them, which have taken a position similar to that

Study	Geography	What policy is the TI attempting to influence?	Tactics used	Arguments used
			within the new incoming conservative Liberal party to stop the regulations becoming law. The NSW Liberal Premier (and future BAT Australia chairman) Nick Greiner was one who was targeted. PM's then international president Australian ex-patriot Bill Murray, wrote ''as an Australian'' to Greiner requesting that he bring ''a sense of balance and common sense back into the regulation of tobacco in Australia'' and maintain the existing warnings.<br><br>In 1992, a lobbyist was ''employed, gaining support of such allies as Business Council of Australia, Confederation of Australian Industry, media, unions, advertising, growers, suppliers''. The Victorian Premier received over 35 000 letters that year, presumably initiated by the industry.<br><br>In 1992, in response to the CBRC's [Centre for Behavioural Research in Cancer] report proposing third generation warnings, the industry commissioned a series of critiques of the CBRC report through its lawyers. With these as ammunition, on 18 June 1992, the TIA filed legal proceedings against the Anti	advocated for Australia''. Henry Goldberg also drew on bad-for-business arguments when writing to Kennett stating: ''…[the warnings proposal] …imposes on cigarette manufacturers an extremely burdensome and expensive regime for the packaging of their products which is without precedent. It constitutes one of the most egregious attacks upon commercial freedoms of business in Australia… Victoria has an obligation to a business such as PM, which is contributing substantially to the economy of this State, to ensure that its commercial freedom is safeguarded.''<br><br>In 1990, a ''questions and answers'' sheet for use by PM lobbyists rehearsed answers to ''Why don't the companies provide additional information and warnings about constituents on the cigarette package labels?'' suggesting that the proliferation of warnings on

Study	Geography	What policy is the TI attempting to influence?	Tactics used	Arguments used
			Cancer Council of Victoria (authors of the CBRC report on labelling) seeking declaration that the report was misleading and deceptive and seeking an injunction to prevent its further distribution.	consumer goods could cause ''warning overload''—that is, a tendency on the part of consumers to begin to ignore warning labels entirely. And that ''one study found that consumers thought products with rather elaborate warning labels were safer than those without them''.<br><br>In Murray's letter to Greiner in 1992, he insisted that the warnings would be ''a defacement of the cigarette package and trademarks''<br><br>Industry law firm Clayton Utz commissioned a national poll about smokers' ''awareness of health warnings about smoking'', known as the ANOP study after the market research company that conducted the interviews. The study asked respondents three questions ''seeking spontaneous health associations with smoking'', including two which probed specifically for health risks (''have

Study	Geography	What policy is the TI attempting to influence?	Tactics used	Arguments used
				you heard, read or seen anything about smoking and health?'' and ''Can you think of anything at all that you have heard about smoking and health?''). Not surprisingly, 99.7% of the sample, under such questioning, were able to say that they had ''heard'' something about smoking and health. This exercise produced a predictable result that allowed the industry to support its claim that awareness of the harmfulness of smoking was all but universal, thus negating the need for new warnings.
Cohen et al, 2001 [11]	USA	Tobacco internet sales; New York State passed a law banning internet, mail, and telephone cigarette orders from being sent directly to consumers, which was scheduled to	Brown & Williamson Tobacco challenged the law… and a federal judge ruled that the law did violate the commerce clause of the US Constitution.	Brown & Williamson Tobacco challenged the law claiming that "The Constitution prohibits any one state from regulating avenues of national commerce such as the Internet . . ."

Study	Geography	What policy is the TI attempting to influence?	Tactics used	Arguments used
		take effect on 1 January 2001.		
Dearlove and Glantz, 2000 [12]	USA (New York)	1. 1990 anti-tobacco campaign including vending machine ban, ban on advertising on taxis, buses, subways, pro-health advertising etc<br>2. Intro 2-A / Tobacco Product Regulation Act (1991) – including requirement of one public health message for every four	**1**<br>Philip Morris Chairman and C.E.O. Hamish Maxwell responded to the announcement with a biting letter to Dinkins [New York mayor]…	**1**<br>Philip Morris Chairman and C.E.O. Hamish Maxwell responded to the announcement with a biting letter to Dinkins [New York mayor], stating, "The financial and other support which Philip Morris has given to public and other institutions in this city appears to me to be acknowledged by the city, if at all, as grudgingly and ungraciously as possible". Maxwell concluded with an implied threat to move Philip Morris headquarters from the city: "It could be reasonably concluded that your Administration has decided that Philip Morris' continuing presence in New York City is one of the city's dozens of embarrassments that it can do without"<br><br>**2**<br>[The industry] worried that the costs associated with displaying an anti-smoking ad would discourage leasing

Study	Geography	What policy is the TI attempting to influence?	Tactics used	Arguments used
		tobacco advertisements in or on municipally-owned or leased property and transportation facilities		agents from accepting cigarette advertisements unless the manufacturer volunteered to absorb the additional costs.
DiFranza and Godshall, 1996 [13]	USA	In 1992, Congress enacted legislation to encourage states to adopt restrictions on the sale of tobacco to minors	The industry's opposition to a requirement that states *enforce* their laws is reflected in the actions of congressman Thomas Bliley… Bliley represented a district in which Philip Morris was the largest employer, and he received $111,476 in tobacco industry contributions from 1985 to 1994.<br><br>The Tobacco Institute and congressman Bliley argued to the DHHS that, because the word "significantly" does not appear in the final language, the DHHS cannot issue regulations that would require states to make significant progress in reducing illegal sales.	The tobacco industry's public stance is that they are opposed to children smoking and are actively discouraging the sale of tobacco to minors. According to Yancey W Ford Jr, executive vice president of the RJ Reynolds Tobacco Company: "the most important factors influencing youth smoking are peer influences, the example of family members and the accessibility of cigarettes – better voluntary enforcement of minimum-age purchase laws currently on the books can reduce youth smoking by at least half".
Epps-Johnson et	USA (Iowa)	Vending machine	A Tobacco Institute report entitled, "1991 Legislative Analysis: Iowa" explained that a	

Study	Geography	What policy is the TI attempting to influence?	Tactics used	Arguments used
al, 2009 [14]		legislation	preemptive vending machine law was necessary in order to "stop a hodge-podge of local ordinances and to prevent the State Legislature from enacting a total vending machine ban." … The Tobacco Institute was successful in achieving their ideal legislative outcome by co-opting House File 232, which was substantially weakened through industry-favorable amendments.	
Freeman et al, 2008 [5]<br><br>(overlaps with [4] and [6])	Transnational	Plain packaging		The industry denies that packaging has an impact on consumption. For example, the Tobacco Institute of New Zealand argued 'package stimuli, including the use of trademark, are of no interest to people not already within the market for that specific product'.<br><br>The tobacco industry also attempted to complicate the issue by suggesting that tobacco control agencies were unclear about what plain packaging would require. Arguments that a 'laundry list' of items had been suggested by 'packaging non-professionals' were presented as an unworkable barrier

Study	Geography	What policy is the TI attempting to influence?	Tactics used	Arguments used
				to implementation.<br><br>They also argue that plain packaging would set a dangerous precedent for other products, such as those containing high amounts of sugar, chocolate, fat or additives.
Gilmore et al, 2006 [15]	Uzbekistan	1994 Health Decree 30: aim to ban tobacco advertising and smoking in public places and introduce health warnings	Within 24 hours [of learning about the health decree] BAT had coordinated counter arguments from its corporate affairs and smoking issues teams and met with Mr Mahsudov from the Cabinet of Ministers. It then met Mr Iskandarov and other health officials a few days later.<br><br>Since Mr Iskandarov refused to withdraw the decree, BAT sought extensive amendments. An amended decree was rapidly circulated alongside highly confidential briefing notes… An order to be issued by Mr Djurabekov on the Cabinet of Minister's behalf, requiring the Ministry of Health to amend decree 30 was faxed from BAT's Tashkent office. Documents suggest it may have been drafted by BAT. It incorporated BAT's main concerns, with the tar and nicotine limits and the bans on	BAT sought to counter each section of the decree, repeatedly claiming to be a responsible manufacturer of a legal product and making three key assertions. Firstly, BAT depicted the decree as jeopardising foreign investment in Uzbekistan, while warning the health ministry that it would lead to "the immediate demise of the domestic cigarette industry" and threaten an investment supported by Karimov. Secondly, BAT refuted the health effects of smoking as accurately described in the decree, suggesting an ongoing controversy in which "smoking has not been proven to actually cause" diseases. Thirdly, the company portrayed Mr Iskandarov's intended restrictions as "seriously

Study	Geography	What policy is the TI attempting to influence?	Tactics used	Arguments used
			smoking in public places, filterless cigarettes, and advertising all cancelled. The advertising ban was replaced with a new code, which seems to be an even less restrictive version of the Russian voluntary code.	interfering with...commercial freedom" and denied that advertising affected consumption: "World wide experience consistently shows that advertising bans do not reduce consumption. Advertising a mature product like cigarettes is not intended to increase the overall market but to expand company market share." Additionally, BAT portrayed Russia's recent voluntary code as epitomising the industry's responsible approach in working with governments to agree adverting standards.
Goldberg et al, 2006 [16]	USA	General marketing regulations/ restrictions		Regardless of the amount spent on advertising, the argument advanced here is that advertising does not influence consumer behaviour, or at most, plays only a small role in the ''marketing mix.''<br>Q. ''… [I]s advertising in the scheme of all the influences… a strong force, a weak force or somewhere in between with respect to influencing consumer

Study	Geography	What policy is the TI attempting to influence?	Tactics used	Arguments used
				behavior?’’<br>A. ‘‘When we take all of the … factors that are inputted into that decision [whether to smoke], it ends up being a relatively weak force.’’ ‘‘There are many things that go into the effect [of inducing smoking initiation]….One of these is communication and a small part of communications [is] advertising.’’<br>Q. ‘‘Would you agree that advertising is a very powerful and persuasive medium?’’<br>A. ‘‘No.’’<br><br>Denial of targeting youth; insistence that only those aged 21 and over are targeted<br>‘‘Philip Morris does not market or sell and has no intention to do so, does not do so, and actually takes many affirmative steps to make sure that it doesn’t happen, sell to either nonsmokers or to minors.’’<br>Q. ‘‘In the entire period of time you

Study	Geography	What policy is the TI attempting to influence?	Tactics used	Arguments used
				were involved with the Camel campaign, Mrs. Beasley, did it ever include among its demographics people under the age of 18?'' A. ''No.'' Q. ''Did it ever do research on nonsmokers or people under the age of 18?'' A. ''No, we did not.'' <br>The tobacco firms meet or exceed existing regulations. It's part of our corporate culture Q. ''Is that [Voluntary Cigarette Advertising and Promotion Code] a code that Philip Morris maintained in its records and followed and observed?'' A. ''Absolutely. I mean, it's more than a document; it's the principle and system under which we marketed.… Philip Morris on its own in recent years has added more to this.'' Q. ''Let me go to the last [point in Philip Morris' mission statement]

Study	Geography	What policy is the TI attempting to influence?	Tactics used	Arguments used
				… Meet or exceed the legal and regulatory requirements that govern our products and business. Why was that important to put in your mission statement, sir?’’<br>A. ‘‘We have a product that’s associated with harm. We have to make sure that we’re within all the laws. And, frankly, we need to be pushing the envelope relative to regulation and legal issues, to make sure that, at a minimum, we’re meeting them; but in a case like the MSA, we’re exceeding them.’
Hammond, 2009 [17]	Transnational	Plain packaging	The regulation was amended … and the legal challenge was not continued.	Tobacco companies also challenged picture warnings in Belgium, where they argued against the feasibility of implementing the 42 required warnings within the proposed time period.
Hammond, 2010 [18]	Transnational	Plain packaging		The tobacco industry has taken a strong stance against “plain” packaging regulations. Philip Morris has characterized plain packaging as “an extreme and disproportionate

Study	Geography	What policy is the TI attempting to influence?	Tactics used	Arguments used
				measure." Japan Tobacco International has also indicated its "categorical" opposition to plain packaging, adding that it would be "disproportionate" and may even be "counterproductive" as a tobacco control measure. British American Tobacco has also stated that, in response to a plain packaging proposal, "we would take every action possible to protect our brands, the rights of our companies to compete as legitimate commercial businesses selling a legal product, and the interests of our shareholders." The industry's primary arguments against plain packaging relate to insufficient evidence that plain packaging would reduce smoking, and intellectual property rights and issues of international trade.
Hiilamo et al, 2012 [19]	Transnational	Health warnings on packs	In the late 1990s, the Canadian government health agencies, non-governmental organisations, and health professionals began campaigning to increase the size of warning labels and include strong photographs	

Study	Geography	What policy is the TI attempting to influence?	Tactics used	Arguments used
			depicting the damage caused by tobacco. The Canadian Tobacco Manufacturers Council (CTMC) tried to stop the proposed regulations by polishing the industry's public image and launching a major lobbying programme. CTMC recognised that 'the Industry's agenda is simply not compatible with that of Canadians' because of the public 'perception that the Industry does not tell the truth' about the health risks, and 'markets its products to youth or, at least, does nothing to discourage it.' In summer 1999, CMTC mounted an advertising campaign claiming that the industry needed to be heard on issues concerning packaging and youth smoking. The industry launched what a 1999 memorandum in PM files described as a 'full blown government relations programme' to lobby government agencies, including 'Revenue, Treasury Board, Finance, Justice, Trade departments and Ministers' offices and the Standing Committee on Health members by CTMC representatives and individual companies.	
Hoek et al, 2012 [20]	New Zealand	Smoke-Free Environments (Controls and		As well as arguing that they have a right to display their 'legal products', tobacco companies have adduced

Study	Geography	What policy is the TI attempting to influence?	Tactics used	Arguments used
		Enforcement) Amendment Bill (2010). The bill proposes removal of tobacco retail displays.		four further arguments against removing Point of sale (POS) displays. They claim that such a policy would reduce staff security (as staff would turn their back on customers to retrieve tobacco products), inconvenience staff, result in lost revenue and increase compliance costs. Collectively, they allege that these outcomes will be disproportionately negative relative to the likely reductions in smoking prevalence.<br><br>Philip Morris has argued that retail staff would be seriously inconvenienced if these products were difficult to access, while customers would allegedly become frustrated by increased transaction times.<br><br>In addition to losing revenue, tobacco companies and interest groups have argued retailers would find the costs of alternative storage units prohibitive. The New Zealand Association of Convenience Stores

Study	Geography	What policy is the TI attempting to influence?	Tactics used	Arguments used
				estimated that removing Point of sale (POS) displays would cost retailers between $45 and $51 million, with individual retailer costs between $8000 and $14 000.
Ibrahim and Glantz, 2003 [21]	USA (California)	AB 221: increase the minimum age to purchase tobacco, as well as all related advertising and promotion restrictions, from 18 to 21 years.		The opponents<br>argue that bill is not consistent with federal law and that it will harm retailers and the state by shifting tobacco sales and the associated tax revenues to neighboring states
Kennedy et al, 2011 [22]	USA (Florida)	1. SB 434 and HB 555 to Regulate Tobacco Product Placement (2000)<br>2. SB 1902 (2002) regarding minimum	**1**<br>In opposition to the bill, testimony was heard from the Florida Retail Federation (a strong tobacco industry ally in Florida), the Petroleum Institute, Gate Corp, S & S Stores, Tom Thumb Stores, and the Tobacco Wholesalers.<br><br>**2**<br>While on the Special Order Calendar, Senator Diaz de la Portilla (R, Miami, $6,578) a smoker	

Study	Geography	What policy is the TI attempting to influence?	Tactics used	Arguments used
		wage payments (TI filed and unrelated amendment)	with an established relationship with the tobacco industry, filed an amendment to the bill which would have granted sole authority to regulate the display and marketing of cigars, fine cut tobacco, and pipe tobacco by retailers to the Division of Alcoholic Beverages and Tobacco.	
Lambert et al, 2004 [23]	Japan	General marketing regulations	In 1994 the tobacco industry also strengthened its self enforced tobacco industry advertising codes to forestall legislation: ‘‘modification of voluntary codes may enable [us] to avoid the legislation of ad restrictions.’	
Landman et al, 2002 [24]	Transnational (mainly USA)	General youth restrictions	In 1991 Philip Morris restated that the success of the "youth initiatives" would be determined by whether they led to a "reduction in legislation introduced and passed restricting or banning our sales and marketing activities" as well as "passage of legislation favorable to the industry" and "greater support from business, parent and teacher groups."<br><br>Philip Morris also sought to enact legal age limits for cigarette sales to deflect blame from itself for youth smoking, saying that such age limits “signal [that] the ultimate responsibility	Philip Morris used its Action Against Access youth program as part of its argument that the FDA's proposal was unnecessary. A 1996 RJR press release argued that the FDA regulation was unnecessary because the industry's We Card program was "now making a measurable difference”.

Study	Geography	What policy is the TI attempting to influence?	Tactics used	Arguments used
			[for youth smoking] belongs to parents and society" rather than tobacco companies. As in the United States, Philip Morris also sought to enact legal age limits [in the EEMA region] to "eliminate anti-tobacco groups' demands for ad bans on the basis of "protection of youth.<br><br>A 1996 RJR press release argued that the FDA regulation was unnecessary…	
Lee and Glantz, 2001 [25]	Switzerland	A referendum on the tobacco and alcohol advertising bans were expected to take place late in 1993 or early in 1994	As part of its effort to oppose advertising restrictions, the tobacco and advertising industries organized a “public hearing” to generate negative press for the idea of advertising restrictions.<br><br>Our [PM] Corporate affairs strategy is to fight both the initiative and the Federal counter-project by offering a reasonable alternative to authorities and Swiss voters through the voluntary advertising restriction agreement came into force on September 1, 1992	
Ling et al, 2010 [26]	USA	Mid-1990s proposal for the Food and Drug Administration (FDA) to regulate	In early 1993, Philip Morris became a founding member of the American Coalition for Entertainment and Sports Sponsorship (ACESS)… which included RJR, US Tobacco, Penske, and International	

Study	Geography	What policy is the TI attempting to influence?	Tactics used	Arguments used
		tobacco, which included restrictions on sport sponsorships	Speedway Corporation… The organization presented itself as a broad coalition of sport sponsors and event promoters, but internally, the stated goal of the organization was ''to defend tobacco sponsorship.'…The group opposed a mid-1990s proposal for the Food and Drug Administration (FDA) to regulate tobacco, which included restrictions on sport sponsorships, by encouraging sports fans to write letters to the FDA against this legislation and sponsoring studies showing public support for corporate sport sponsorship.<br><br>In 1996, Phillip Morris and US Tobacco proposed an alternative plan to the proposed FDA ban on sports sponsorship that would have allowed continued sponsorship of rodeo and motor sports. The tobacco companies filed lawsuits challenging the FDA proposal.	
MacKenzie et al, 2004 [27]	Cambodia	General marketing regulations:<br><br>A 1991 regional assessment reported: "[n]o	The unlimited scope for advertising and promotional activities was a key part of the appeal of the Cambodian market. A 1991 regional assessment reported: "[n]o known restrictions in any form for Above-the-line or Below-the-line activities. Absence of any form of Government policy concerning	

Study	Geography	What policy is the TI attempting to influence?	Tactics used	Arguments used
		known restrictions in any form for Above-the-line or Below-the-line activities. Absence of any form of Government policy concerning tobacco issues, advertising and promotion." BAT was predictably anxious to preserve this unrestricted marketing environment.	tobacco issues, advertising and promotion." BAT was predictably anxious to preserve this unrestricted marketing environment.<br><br>An industry initiated programme of voluntary health warnings was developed for brands across Indochina, intended to "demonstrate that the industry is taking a responsible attitude in markets where there is no legal requirement for health warnings on product".	
Mackey et al, 2013 [28]	Uruguay	Plain packaging		Philip Morris, the world's largest tobacco company, has challenged Uruguay's and Australia's laws under bilateral investment treaty (BIT) obligations. Philip Morris alleges that Uruguay's regulations violate

Study	Geography	What policy is the TI attempting to influence?	Tactics used	Arguments used
				provisions of a Switzerland---Uruguay BIT, which prohibits foreign investors from being subjected to unreasonable treatment under “fair and equitable treatment” provisions.
Mamudu et al, 2008 [29]	Transnational	FCTC	British American Tobacco brought the 3 major tobacco companies together to develop a worldwide voluntary tobacco industry regulatory regime [2001 International Tobacco Products Marketing Standards; ITPMS).<br><br>Tobacco companies perceived youth smoking prevention as a way to "make a significant gesture that would divert attention from the FCTC, moderate the WHO's moves toward the FCTC and bring the tobacco companies together against the FCTC.<br><br>After the ITPMS was announced, Cerberus Group officials worked to raise public awareness of it, calling on governments, United Nations agencies, and the World Bank to have "faith in a 'new [tobacco industry] initiative’”.	British American Tobacco considered the WHO FCTC public hearings in October 2000 very important, and mobilized allies to make submissions… The Cerberus Group companies' submissions focused on the industry's ability to regulate itself, the right of adults to choose to smoke, and the idea that youth smoking prevention should be the focus of the FCTC.<br><br>On June 15, 2001, the group finalized the ITPMS. The communication plan, “key external messages” and the questions and answers the Cerberus Group developed for the ITPMS conveyed the public message that the tobacco industry could regulate itself and that smoking should be limited to adults.

Study	Geography	What policy is the TI attempting to influence?	Tactics used	Arguments used
Mandal et al, 2009 [30]	European Union	European Union's Tobacco Products Directive (2001/37/EC)	Detailed strategies for contesting the directive were developed carefully both by individual companies and various pan-European membership organisations, with lobbying targeted according to the audience and the stage of the directive's legislative passage.<br><br>Tobacco House was a venture spearheaded by PM, Societé Nationale d'Exploitations Industrielle des Tabacs et Allumettes (SEITA), International Union of Tobacco Growers, Federation Europeenne des Transformateurs de Tabac (FETRATAB) and Confederation Europeenne des Detaillants en Tabac (CEDT) (but notably, not BAT) to bring together a far broader group of tobacco sector interests than present in existing pan European organisations, a move seen as strategically advantageous at a time when the industry was under pressure.<br><br>The unions, who were to be told that manufacturers were being "forced" to consider relocating production, played a key role in lobbying, particularly in presenting economic arguments, generating press interest and enlisting political support. The	In the UK, press releases highlighted (and local and national media coverage subsequently focused on) the alleged job losses associated with the TPD.<br><br>He [BAT's lawyer] pushed for the Committee to state that the proposed directive was unconstitutional and that legislation should await the ECJ judgement on the advertising ban.<br><br>BAT's Chairman and Director of Corporate and Regulatory Affairs sent letters voicing their concerns to the Secretary of State for Health, Alan Milburn and the Permanent Secretary, Chris Kelly. In line with BAT's efforts to re-position itself as a responsible company, these letters stressed that BAT was in favour of sensible regulation, but claimed the directive was "far from sensible", highlighting concerns about the directive's legality, impacts on jobs and ability to achieve health

Study	Geography	What policy is the TI attempting to influence?	Tactics used	Arguments used
			UK Tobacco Workers Alliance (TWA), a cooperative of several trade unions, was described as “unrelenting in their [sic] efforts” to lobby against the directive, and, along with other unions, played a role in organising protests against the directive. The Transport and General Workers Union and Britain’s General Union also played a role…At an EU level the industry managed to obtain support from the European Committee of Food, Catering and Allied Workers Unions, which issued a press release highlighting the “massive negative social effects on employees in the tobacco sector“….Public relations formed a key component of the industry’s strategy and plans to use the media at local, national and European levels, with a focus on employment aspects, were successfully implemented. In the UK, press releases highlighted (and local and national media coverage subsequently focused on) the alleged job losses associated with the TPD, rather than any potential health gains… BAT also resorted to placing its own articles, advertisements and letters within the media to generate further coverage.	benefits.<br><br>Once the product content and labelling provisions appeared settled, BAT appealed for a longer transition period…. [BAT] claimed the technical requirements, including additional testing, yield reductions and new labelling, would impose financial costs on business that would threaten the viability of smaller firms.<br><br>They established pan-industry working groups to develop argumentation against key aspects of the proposals and collectively lobbied against them claiming, for example, that there was no rationale for such European tobacco control measures, which were instead a matter for Member States, and that the legal basis was suspect.<br><br>Initially, in response to the 1998 consultation, the industry tried to negate the need for further

Study	Geography	What policy is the TI attempting to influence?	Tactics used	Arguments used
			A tripartite meeting in July 1998 to discuss the proposed directive brought together representatives from DG SANCO, health organisations and the industry. CECCM's view of the meeting was that the health representatives did not believe the industry and challenged their integrity. After the event, Paul Sadler of BAT, wrote to Dr Hunter (Director of DG SANCO) to express his disappointment in the meeting and his belief that tobacco company views were not appreciated.<br><br>Byrne rejected the economic and employment claims made by BAT, a fact which chairman Martin Broughton claimed to find astonishing, warning Byrne in a letter that the draft directive: "deserves to be challenged in the Courts".<br><br>Lobbying of such officials occurred through two routes: general meetings between industry and DG SANCO members, and separate technical consultations on scientific and technical elements of the directive. The agreement to hold these technical consultations on four key topics, (yields,	legislative measures by claiming that harmonisation had already been completed in the areas of tar yields and labeling.<br><br>A number of arguments were raised in relation to economic impacts. First, the threat to employment and balance of trade within the EU was raised, with the directive being described as an "own goal for the EU economy" that would drive jobs and investment outside the Union. Various figures were produced and widely used in lobbying including a claim that in the UK alone up to 8,300 jobs were at risk. Others claimed that 9,000 tobacco manufacturing jobs in the EU and at least a further 30,000 jobs in companies supplying tobacco manufacturers were at risk. Second, BAT argued that the directive would particularly disadvantage Europe-based manufacturers operating in international markets, claiming that the TPD would involve "handing a

Study	Geography	What policy is the TI attempting to influence?	Tactics used	Arguments used
			labelling, descriptors, and additives, measurement methods and toxicological testing), had been secured at a meeting between industry representative and DG SANCO officials on 23rd November 1999 and was described by the industry as "a major step forward" which would enable them to engage the Commission in detailed discussion on the directive.<br><br>The VdC [German tobacco manufacturers association] had sympathetic German contacts in Kurt Lechner, rapporteur of the Legal Committee and Werner Langen, rapporteur for the Industry Committee… As members of a group of 39 MEPs, they tabled several amendments to the directive including a deletion of the export requirement, a smaller size and weaker specification for the health warnings, and the use of misleading descriptors in most circumstances, all key changes the industry desired.<br><br>BAT instead produced its own economic impact study, focusing on potential job losses in the UK. Entitled "Have you ever felt unfairly singled out?"… conveyed a message that the	competitive advantage to the world's largest cigarette manufacturer, US-based Philip Morris".<br><br>BAT instead produced its own economic impact study, focusing on potential job losses in the UK. Entitled "Have you ever felt unfairly singled out?"… conveyed a message that the TPD would bring significant job and trade losses whilst providing no health gains. Indeed, the report claimed the directive would "wipe out" UK tobacco manufacturing and included headline figures which claimed that around 8,300 jobs were at risk at just two sites, even though the small print showed that the total employment in BAT's UK factories was only 1,565<br><br>Despite inconsistencies in the legal advice and having received at least one clear indication that the directive was consistent with EU law, BAT and others acting on its behalf,

Study	Geography	What policy is the TI attempting to influence?	Tactics used	Arguments used
			TPD would bring significant job and trade losses whilst providing no health gains. Indeed, the report claimed the directive would “wipe out” UK tobacco manufacturing and included headline figures which claimed that around 8,300 jobs were at risk at just two sites, even though the small print showed that the total employment in BAT’s UK factories was only 1,565…The UK economic impact study published jointly by BAT and several trade unions in September 2000, stimulated written questions to the Commission from MEPs, such as Glyn Ford (UK Labour Party), on whether the Commission itself had carried out a comprehensive assessment.<br><br>In 2000, before the directive had passed into law, BAT filed an access case to the Court of First Instance, requesting access to the international scientific research the Commission had considered and based its proposals on.<br><br>The Commission did eventually append a brief impact assessment form to their draft TPD proposal in 1999 which fulfilled its obligations, given that at that time, only limited guidance	notably Scott Crosby of the legal firm Kemmler Rapp Böhlke, quickly sought to assert that the directive was incompatible with the EU Treaty.<br><br>Looking to replicate successful industry campaigns that used trade arguments to prevent the adoption of plain packaging regulation in Canada and critically undermine provisions for ingredients disclosure in Thailand, BAT generated a legal analysis that depicted the TPD as potentially breaching WTO requirements, which could therefore expose EU member states to potentially costly disputes.

Study	Geography	What policy is the TI attempting to influence?	Tactics used	Arguments used
			on undertaking impact assessments existed (interview with European Commission official, September 2008). Perhaps unsurprisingly (given that the impact assessment the Commission produced was supportive of the TPD proposals), BAT and its representatives were not satisfied and labelled the assessment as "abysmal" and "wholly misleading".	
McDaniel and Malone, 2005 [31]	USA	Since 2000, Philip Morris (PM)...has been aggressively pursuing US Food and Drug Administration (FDA) regulation of tobacco products - it came to regard ''reasonable'' regulation as a way to end its isolation and redefine the company as socially	PM also explained both publicly and privately that it regarded some sort of regulation as inevitable. The company [PM] predicted that the Republican party controlled Congress of 2001 was more likely to enact what it regarded as ''reasonable'' regulation than any Democratic party controlled future Congress… PM argued that it was better to act now than to risk more onerous regulations.<br><br>PM's law firm Arnold and Porter had written several drafts of model legislation. PM had also provided members of Congress with bill language.<br><br>To achieve its goal of ''tough'' but ''reasonable'' regulation, PM employed a number of different strategies. First and	PM's white paper also advocated the continued use of product descriptors like ''light'' and ''ultra light''. In November 2001, the National Cancer Institute (NCI) released a comprehensive report showing that despite the tobacco industry's marketing claims, light or low tar cigarettes did not reduce health risks. In response, the major health groups (ACS, AHA, ALA, and CTFK) called on tobacco companies to eliminate these terms from cigarette packages and advertising. PM refused, asserting in a press release that, for smokers, such descriptors ''serve as useful points of comparison for cigarette brands

Study	Geography	What policy is the TI attempting to influence?	Tactics used	Arguments used
		responsible	foremost, company representatives worked with legislators, meeting with staff to explain PM's views, helping to write legislation, and lobbying on behalf of legislation PM supported. Initially, company representatives worked most closely with Republicans, who PM saw as more inclined to produce acceptable legislation.<br><br>PM also engaged in an extensive public relations campaign to advance its goal of ''reasonable'' regulation. PM executives and consultants wrote op-eds and letters to editors of both major and local newspapers, participated in editorial board meetings, gave interviews to journalists, and had speaking engagements at local community organisations such as Rotary and the Chicago Mexican American Chamber of Commerce.<br><br>PM's media campaign had a grassroots element as well. PM mobilised its field action teams, lobbyists and consultants in all 50 states whose job was to enlist supporters for a variety of PM causes. In a three month period, team members met with representatives of 650 different organisations, including	regarding strength of taste and reported tar yield''.<br><br>In terms of marketing restrictions, PM advocated codifying those contained in the MSA, which limited tobacco advertising seen by children; however, the company rejected restrictions on marketing seen predominately by adults. The company wanted to continue communicating with what it termed ''verified'' adult smokers through direct mail, events in adult only facilities, and over the internet.

Study	Geography	What policy is the TI attempting to influence?	Tactics used	Arguments used
			hospitality, beverage, grocery, retail, and convenience store associations, wholesalers, chambers of commerce, and one health organisation (the Utah Nurses Association). At these meetings, team members briefed organisation representatives on PM's position on FDA regulation and asked for an official show of support—by contacting members of Congress, writing letters to local newspapers, or asking the national organisation to endorse FDA regulation of tobacco.<br><br>PM also communicated its views…through individual letters, one-on-one and group meetings, and mass mailings. The mailings might include PM's white paper on FDA regulation, a question and answer document, a fact sheet on the MSA, or copies of op-eds by PM executives. In June 2001, the company launched Tobacco Connections, a newsletter devoted to tobacco policy issues of importance to tobacco growers. The inaugural issue, mailed to 130 000 farmers, was devoted to explaining PM's position on FDA regulation and how such regulation would benefit growers.	

Study	Geography	What policy is the TI attempting to influence?	Tactics used	Arguments used
			In a further effort to claim the ''reasonable'' middle ground of the debate, PM portrayed opponents of what it regarded as moderate legislation as extremists or obstructionists. In an August 2001 interview in The Wall Street Transcript, PM lawyer Mark Berlind expressed surprise at some public health groups' opposition to the Frist and Davis bills. He asserted that these groups, in supporting only ''the most radical, extreme kind of medical product regulation'' were working against the passage of reasonable and effective legislation.	
McDaniel and Malone, 2009 [32]	USA	General marketing regulations	PM's corporate affairs executive Jeannine Dowling asserted in 1984 that ''women might be…our natural enemies'' due to their negative feelings about tobacco…<br><br>Another benefit of tobacco company contributions to women's organizations was the creation of allies who could be called upon to defend the industry from regulation…. That same year [1990], at the "behest" of PM and "out of gratitude" for the company's support, American Women in Radio and Television wrote letters to Congress opposing a bill to limit cigarette advertising.	

Study	Geography	What policy is the TI attempting to influence?	Tactics used	Arguments used
			If women's organizations could not be secured as outspoken allies, PM regarded it as important to ''freeze'' them in neutral mode so that they did not support policies, such as cigarette advertising bans, that conflicted with tobacco company business objectives…Funding women's organizations was regarded as key to "neutralizing" them.<br><br>When a funded organization spoke out against PM, continued funding could be jeopardized, as was evident in 1999, when the national Young Women's Christian Association (YWCA), whose local chapters had received more than $70,000 in PM grants that year, signed a public letter calling on the company to change its cigarette marketing practices. PM's vice president of corporate affairs, Ellen Merlo, noted in an internal e-mail that ''[W]e certainly do not require that worthy recipients speak out for us, but if they are publicly against us, [I] would give thought to continued support''	
Mekemson and Glantz, 2002 [33]	USA (Hollywood, California)	Promotion of tobacco through entertainment	The cigarette companies claimed to solve the problem by modifying their voluntary Cigarette Advertising and Promotion Code. In	

Study	Geography	What policy is the TI attempting to influence?	Tactics used	Arguments used
		media	1990, they added: “No payment shall be made by any cigarette manufacturer or any agent thereof for the placement of any cigarettes, cigarette packages, or cigarette advertisements as a prop in any movie produced for viewing by the general public”.	
Nakkash and Lee, 2009 [34]	Lebanon	Marketing restrictions and health warnings	The industry supported a voluntary code in Lebanon to delay or prevent binding regulation.<br><br>Documents suggest intense industry lobbying again delayed and weakened the proposed revision [1992, PHC began discussing stronger warnings which would be rotated, occupy 20% of pack size and extend to broadcast media]: ‘‘[f]ollowing discussion with the tobacco companies and advertising agencies, the government has made no moves to introduce it. Industry/government talks on an ongoing informal basis aim to slow down the possible introduction of further legislation’’.	
Neuman et al, 2002 [35]	Europe (mainly Germany, UK, Netherlands, Denmark)	Through Council Directive 98/43/EC, the European Community (EC) sought to end	**Germany**<br>Philip Morris sought to maintain strong political alliances within Germany, and to provide German legislators with a variety of arguments against the advertising ban…. Industry documents reveal that the German	**Germany**<br>A 1993 Philip Morris marketing freedoms presentation describes Philip Morris’s proposals to defeat the EC advertising ban though cooperation with Kohl: “Use all

Study	Geography	What policy is the TI attempting to influence?	Tactics used	Arguments used
		all tobacco advertising and sponsorship in EC member states by 2006. Initially proposed in 1989, the directive was adopted in 1998, and was annulled by the European Court of Justice in 2000 following a protracted lobbying campaign against the directive by a number of interested organisations including European tobacco companies. A	tobacco industry viewed former German Chancellor Helmut Kohl as a close ally from as early as 1978.<br><br>Industry documents suggest that the industry worked with Germany to introduce a weak proposal designed to replace the proposed, stronger EC advertising ban. According to industry documents, this proposal was drafted by the tobacco industry and was intended to be submitted, without acknowledgment of its true origin, through German representatives to the EC. The proposal was produced by the Confederation of European Community Cigarette Manufacturers (CECCM), an organisation that the industry created to coordinate political efforts of all major EC tobacco manufacturers.<br><br>**UK**<br>In 1990, UK Prime Minister Margaret Thatcher denounced the EC advertising directive as "meddling" in the domestic affairs of member states. Philip Morris' documents state that in July, 1992, shortly after she left office as Prime Minister, Philip Morris Companies hired Thatcher as a	possible German influence to prevent a weakening of the blocking minority. Work with Chancellor Kohl to put ad ban directive on commission subsidiarity list." The principle of subsidiarity pertains to policy areas in which the EC as well as individual member states may both have competence to act. In these areas, the EC may only take action if the proposed objective cannot be sufficiently achieved by member states. The tobacco industry recognised subsidiarity as a potential basis on which to have the advertising ban abandoned. Kohl's inclusion of the advertising ban on the subsidiarity list would constitute an attempt to veto the ban on the basis of infringing on national sovereignty. Philip Morris claimed success in securing German opposition to the advertising directive on the basis of subsidiarity in a 1993 memo prepared in part by David Greenberg, a Corporate Affairs Executive for Philip Morris in the EEC

Study	Geography	What policy is the TI attempting to influence?	Tactics used	Arguments used
		new advertising directive was proposed in May, 2001.	geopolitical consultant for a fee of US$250 000 per year and an annual contribution of US$250 000 to the Margaret Thatcher Foundation. As reported by The Times (London), a March 31, 1992, memorandum from Geoffrey Bible, executive Vice-President of Philip Morris Companies, described key areas in which Philip Morris sought to use Thatcher's international experience. These included the proposed EC ban on tobacco advertising<br><br>In 1992, Philip Morris also considered Kenneth Clarke, the Secretary of State for Education and Science at the time and Secretary of State for Health from 1988 to 1990, as an ally in opposition to the ban. Panel 3 shows a 1992 correspondence between Clarke and Philip Morris' Ian Sargeant in which Clark assures Sargeant that he will do his best to ensure that the Tory government maintains its opposition to the EC advertising ban. Clarke's links to the tobacco industry have continued to the present. Beginning in 1998, Clarke received UK£100 000 a year to serve as a deputy chairman of British American Tobacco while also holding a seat in the UK House of	region: "the German Government has included the EC ad ban in its list of directives to be dropped out under the subsidiarity principle."

Study	Geography	What policy is the TI attempting to influence?	Tactics used	Arguments used
			Commons.<br><br>**Netherlands**<br>Philip Morris lobbying efforts in the Netherlands employed a strong publicity campaign against the advertising ban. The 1991 Philip Morris-EEC corporate affairs agenda describes a tobacco industry-organised "local coalition of opinion leaders in the Netherlands, 'Multiple Choice', which undertook communications efforts and lobbied the Dutch government on the EC directive."<br><br>**Denmark**<br>Philip Morris sought to preserve Denmark's opposition to the ban through the creation of the Committee for Freedom of Commercial Expression, a group designed to cultivate opposition to the ban in Danish social and governmental circles. The committee was to be "managed at arm's length—distanced from Philip Morris". The Committee thus could have appeared to the public as an independent third party. In building the committee, industry recruited "more than 50 prominent Danes, including a leading	

Study	Geography	What policy is the TI attempting to influence?	Tactics used	Arguments used
			Constitutional lawyer, the President of a major brewery, a leading Danish writer and philosopher and a well-known architect".<br><br>**EU-wide**<br>The tobacco industry established alliances with groups representing various industries on a pan-European level… By 1991, Philip Morris had built coalitions with International Chamber of Commerce (ICC) and the Union of Industrial and Employers' Confederations of Europe, two pan-European lobbying bodies. The 1991 Philip Morris-EEC corporate affairs agenda reports that "the ICC's General Secretary sent letters to key EC officials" in opposition to the proposed advertising directive.<br><br>Philip Morris also activated the communications and business communities "in the defense of marketing freedoms for tobacco", producing position papers encouraging media and advertising groups "to raise their voices" before local governments and the EC.<br><br>Through the Adam Smith Institute (ASI), a	

Study	Geography	What policy is the TI attempting to influence?	Tactics used	Arguments used
			British policy institute based in London, the tobacco industry commissioned two "separate but complementary projects . . . within a total budget of £30,000". These resultant reports would argue against the EC advertising directive by placing "the EC anti-tobacco proposals in the context of a host of proposals which progressively restrict personal freedom".<br><br>As an alternative measure to the EC advertising directive, the European tobacco industry developed a voluntary code of practice for tobacco advertising and sponsorship. On May 12, 1992, Philip Morris' Ian Sargeant described a plan to create "an EC-wide industry code of conduct on advertising that would reassure the politicians that the industry had put its own house in order and would remove the political pressure to legislate". Like the industry draft directive on harmonisation, the voluntary code was produced by CECCM in 1992 and proposed minimal restrictions, primarily prohibiting tobacco advertising and sponsorship "directed towards persons who are under eighteen years of age".	

Study	Geography	What policy is the TI attempting to influence?	Tactics used	Arguments used
O'Sullivan and Chapman, 2000 [36]	China	General marketing regulations	As in the West, self regulating the marketing of cigarettes was used to avoid legislation as well as to portray industry responsibility. "If done honestly and with the concurrence of government authorities—and in advance of restrictive government proposals—industry codes can be accepted instead of legislation." RJR considered the utility of a voluntary code, to demonstrate that: "American tobacco manufacturers adhere to the advertising laws and regulations of the countries in which they operate."	As in the West, the TTCs ran a public line on their declared position of not targeting non-smokers, particularly women and children:"Although some advertisements appeal to young adults who smoke, American tobacco manufacturers do not target any advertisements towards children . . .Moreover, research reveals that smoking initiation is a complex process, and advertising plays only a "miniscule" role—"if any"—in that process."
Outterson, 2011 [37]	USA	The Tobacco Control Act (2009) made three changes to cigarette warnings. First, existing warnings on cigarette packages must be replaced with nine new specified verbal warnings.	The case decided in November, R.J. Reynolds Tobacco Company v. FDA, challenged the second two of these three changes, as implemented through an FDA rule published on June 22, 2011.	

Study	Geography	What policy is the TI attempting to influence?	Tactics used	Arguments used
		Second, nine new graphic images must be paired with the textual warnings on a rotating basis. Finally, companies must move the warnings from the side of the package and devote at least the top 50% of both the front and back panels to the government-mandated messages.		
Pan American Health Organisation, 2002 [38]	Latin America	1. General advertising restrictions in the Latin American region<br>2. 1991 -	**1**<br>As pressure for greater regulation and restrictions grew, the cigarette companies employed diverse resistance tactics. As well as developing alternative marketing and promotional strategies such as brand stretching and nightclub promotions to stay	

Study	Geography	What policy is the TI attempting to influence?	Tactics used	Arguments used
		Caracas, Venezuela: ban on outdoor advertising (tobacco and alcohol)<br>3. 1991 – Honduran proposal for advertising ban	ahead of the law, the companies found an important deterrent to stricter governmental regulations in voluntary marketing codes.<br><br>**2**<br>The companies [BAT and PM] also joined forces to oppose legislation that restricted tobacco advertising. In 1991, when the city of Caracas, Venezuela, passed an ordinance banning all outdoor advertising of cigarette and alcohol, BAT's Bigott and PM's Catana, allying themselves with outdoor advertising agencies, explored legal and other options to counter the ban.<br><br>**3**<br>A successful lobbying effort occurred in 1991 when the tobacco companies and their advertising allies managed to block a Honduran proposal for an advertising ban, gaining agreement from the Minister of Health that the companies themselves would write a new draft of the proposed regulation.	
Physicians for Smoke-Free Canada, 2008 [6]	Transnational (mostly Canada)	1. Canada 1992-4: Tobacco Products	**1**<br>On May 20 1993, Rothmans senior lawyer responsible for regulatory affairs, James Seddon invited his colleagues in the other for-	**1**<br>Their position [was] that the law was beyond federal jurisdiction and that it was inconsistent with Charter-

Study	Geography	What policy is the TI attempting to influence?	Tactics used	Arguments used
(overlaps with [4] and [5])		Control Act<br>2. Australia 1992-5: plain packaging and on-pack health warnings<br>3. Canada 2000: health warnings<br>4. Canada 1997-9: plain packaging<br>5. Finland 1993: proposal to strengthen the 1977 law banning tobacco advertising<br>6. South Africa	profit multinational companies to consider a joint approach to plain packaging and larger health warnings…. By late summer his proposed group had met, and by November had fully taken shape.<br><br>The International Chamber of Commerce (ICC) was among those recruited by BAT into writing a strongly worded letter to Canada's trade minister, opposing plain packaging in Canada on trademark grounds. Despite WIPO's advice to the contrary only a month before, the ICC maintained this would be a serious breach of Canada's obligations under the Paris Convention.<br><br>A month later, with hearings about to begin, McDonald again wrote the Plain Pack group (on April 5, 1994). This time he had more specifics to report on the CTMC's strategy, which closely followed the Plain Pack Group strategic outline:<br>• They worked to prompt an energetic public debate. (Their campaign manager, David Small, coordinated messaging through frequent bulletins and an aggressive media campaign).	protected freedoms of expression.<br><br>The International Chamber of Commerce (ICC) was among those recruited by BAT into writing a strongly worded letter to Canada's trade minister, opposing plain packaging in Canada on trademark grounds. Despite WIPO's advice to the contrary only a month before, the ICC maintained this would be a serious breach of Canada's obligations under the Paris Convention.<br><br>They were not shy to describe their strategy to their multinational Colleagues… To accommodate their Canadian operations, the European headquarters of the Canadian companies followed this strategy and worked behind the scenes to get an object filed by the European Union under GATT technical barriers to trade (TBT) procedures. On May 24, 1993, their efforts paid off when the EU GATT Inquiry point wrote the

Study	Geography	What policy is the TI attempting to influence?	Tactics used	Arguments used
		1994: 25% pack health warnings<br>7. Hong Kong 1996: Smoking Public Health Amendment Bill, restricting advertising and requiring health warnings<br>8. New Zealand 1997: increase size of health warnings<br>9. South Africa 1998: Tobacco	• They created their own body of evidence by publishing their own materials and papers. They soon commissioned market research from Decima and hired university-based researcher, Zalton Amit, to counter the findings of the Canadian Cancer Society).<br>• They created their own experts. (They engaged John Luik coordinate 'academics who would argue against plain packaging,' and engaged former Mountie Rod Stamler to say that plain packaging would lead to contraband).<br><br>This campaign would continue to involve third-party allies. In the first week of July 1994, 30 representatives of "packagers, retailers, growers, printers, suppliers and their workers" were brought together to work on the next steps of the campaign.<br><br>Philip Morris and RJ Reynolds engaged former U.S. trade representative, Carla Hills, and former Deputy Trade Representative, Julius Katz.<br><br>When the Cancer Society appeared before the	Canadian authorities to say that the new 25% warnings were "excessive" (they said the EU warnings which were only of 4 to 6% of the package worked just as well).<br><br>Philip Morris and RJ Reynolds engaged former U.S. trade representative, Carla Hills, and former Deputy Trade Representative, Julius Katz to tell the Canadian Commons Committee that plain packaging would be an "unlawful expropriation" of their trademark rights and that "the compensation claims of affected foreign trademark holders would be staggering, amounting to hundreds of millions of dollars."<br><br>On Thursday, May 14, 1994 the last set of 6 witnesses were heard, and the last word was given to the Canadian Tobacco Manufacturers Council. Plain packaging, they said, would lead to contraband, would make cigarettes cheaper and more

Study	Geography	What policy is the TI attempting to influence?	Tactics used	Arguments used
		Products Control Amendment Bill<br>10. Sri Lanka 1999: proposed National Authority on Tobacco and Alcohol<br>11. EU 2000-1: EU Directive on Tobacco Advertising<br>12. FCTC 2003	committee to offer its research findings, the CTMC countered with a review they had commissioned from Concordia professor, Zalton Amit, which eviscerated the CCS-funded study.<br><br>**2**<br>The companies also approached the vice-consul (commercial) of the British Consul General in Sydney, Peter Hughes to request assistance from the U.K. government in their "dealings with government in Australia" over package reforms. A letter was sent in August 1992, and a meeting was held with him the following April. Mr. Hughes obligingly made an inquiry of the British industry ministry, but the answer he received and forwarded in July 1993 was consistent with virtually all the external advice the companies received: there were no trade barriers to restraining the use of trademarks on cigarette packages. To think otherwise, in the opinion of the British government, would require several "large and imaginative leaps".<br><br>**3**<br>BAT director of Corporate and Regulatory	available to youth, would result in the direct loss of 1,200 jobs, would expose the government to "billions of dollars" of compensation claims and would be an abuse of process, in light of the industry's constitutional challenge to the Tobacco Products Control Act.<br><br>**2**<br>In January 1994, BAT's Australian subsidiary nonetheless took these large and imaginative leaps and told a government commission of inquiry into the tobacco industry that "The Company does not oppose a review of health warnings, only pack design regulations which take no account of registration of trademarks and pack designs, intellectual properties and rights advocated by GATT".<br><br>A year later, in February 1995, BAT's Australian subsidiary, WD & HO Wills, provided the Senate Inquiry into the Tobacco Industry and the Costs of Tobacco Related Illnesses

Study	Geography	What policy is the TI attempting to influence?	Tactics used	Arguments used
			Affairs wrote the EU Commissioner for Enterprise and Information Society, Erkki Liikanen, to protest the new Canadian health warnings.<br><br>**6**<br>"These are serious infringements of valuable property rights which will expose the South African government to legal challenge."<br><br>**9**<br>The response of the Tobacco Institute of Southern Africa to this legislative proposal … Severe embarrassment and even international litigation could result".	with a supplementary submission, focused entirely on generic packaging. This strongly worded submission concluded that plain packaging would violate "the legal and constitutional rights of the manufacturers who own them. Loss of brand rights would lead to substantial claims for compensation."<br><br>**4**<br>The industry again responded with a flurry of trade objections, including a repetition of their claim, against WIPO advice, that the regulations would "expose Canada to legitimate and well-founded complaints under World Trade Organization agreements such as the Agreement on Trade-Related Aspects of Intellectual Property Rights (TRIPS) and the Paris Convention for the Protection of Industrial Property."<br><br>**5**

Study	Geography	What policy is the TI attempting to influence?	Tactics used	Arguments used
				The Finnish Tobacco Manufacturers Association told parliament that "The prohibition against the use of symbols combined with a prohibition to register the trademark of a tobacco product as a trademark for a product other than tobacco product would be incompatible with the fundamental principles of the trademark rights, defined in the Paris Convention binding on Finland."<br><br>**6**<br>In a letter to the health ministry, Philip Morris claimed that the proposed larger health warnings would infringe their property rights. "Protection of International Property Rights has provided assurances to international consumer products companies that their trademark rights will be respected and protected against infringement or expropriation. Yet the proposed regulations, which would obscure 25% of the front package and 50% of

Study	Geography	What policy is the TI attempting to influence?	Tactics used	Arguments used
				the back package, would seriously infringe these trademark rights, causing consumer confusion as to source, weakening brand identification and generally amounting to a government expropriation of these valuable property rights"<br><br>**7**<br>The Tobacco Institute of Hong Kong protested that "The Bill's proposals also would effectively diminish the commercial value of trademarks lawfully registered and used in Hong Kong, without any compensation to the trade mark owner. They may also violate the Paris Convention for the Protection of Intellectual Property and that part of the General Agreement on Tariffs and Trade (GATT) dealing with Trade Related Aspects of Intellectual Property Rights (TRIPS). This would send a powerful message to the international community concerning the respect which Hong Kong has for

Study	Geography	What policy is the TI attempting to influence?	Tactics used	Arguments used
				intellectual property rights”.<br><br>**8**<br>In a submission to the government, the Tobacco Institute of New Zealand charged that the proposals were “an unwarranted and unjustifiable interference with the intellectual property rights of tobacco companies and “contrary to New Zealand's international obligations undertaken in the WTO/TRIPS Agreement which New Zealand has ratified and by which it is legally bound.”<br><br>**9**<br>The response of the Tobacco Institute of Southern Africa to this legislative proposal was to claim that the measures were a technical barrier to trade. “The implementation of this Bill will probably result in a violation of some of South Africa's international obligations… A state cannot escape its international legal obligations vis

Study	Geography	What policy is the TI attempting to influence?	Tactics used	Arguments used
				à vis other states by relying on its domestic law. … Severe embarrassment and even international litigation could result”.<br><br>**10**<br>BAT’s subsidiary, the Ceylon Tobacco Company, challenged the proposed law as raising “serious issues … under a number of international agreements to which Sri Lanka is a signatory, including the Paris Convention for the Protection of Industrial Property, the Agreement on Trade Related Intellectual Property Rights and Agreement on Technical Barriers to Trade.)<br><br>**11**<br>In a meeting with the European Parliament Committee on Environment, Public Health and Consumer Policy, the industry said that because descriptors, like ‘light’ were part of a trademark, and therefore “a prohibition of use of such a combined trademark would

Study	Geography	What policy is the TI attempting to influence?	Tactics used	Arguments used
				violate the TRIPS Agreement and the Paris Convention.” The industry commissioned an extensive argument regarding trade agreement impediments to implementing the directive.<br><br>Japan Tobacco International filed a complaint in mid-September 2001 with the European Court of First instance claiming that the ban on ‘light’ and ‘mild’ was a violation of intellectual property laws.<br><br>**12**<br>British-American Tobacco's Submission to the WHO's Framework Convention on Tobacco Control warned that “ The WHO's proposals to ban tobacco advertising and descriptors such as 'Lights', could infringe commercial and intellectual property rights guaranteed in international law and could clash with provisions embodied in national constitutions protecting freedom of speech.
Sebrie and	Latin America	General	As in the United States, the industry's Latin	

Study	Geography	What policy is the TI attempting to influence?	Tactics used	Arguments used
Glantz, 2007 [39]		marketing regulation	American "educational" programs concentrated on promoting "adult choices"…. Anticipating new regulations on tobacco marketing activities outside the United States. Geoffrey C Bible, the chairman of the board and chief executive officer of Philip Morris, the parent company, asked PMLA, as well as other regional officials of PMI, to prepare a report describing what had been done on the issue of preventing youths' access to tobacco… All PMI regions were requested to demonstrate they had "credible youth smoking prevention] programs in place, and that [they] work cooperatively with governments on the issue,"' In response, in August 1997, Leiber prepared for Bible the "Latin America Report: Youth Access Prevention and Education Programs." … As a public relations tactic to achieve this support, Leiber said, PMLA representatives "regularly meet with presidents, first ladies, prime ministers, ministers of education, ministers of health and presidents of congressional commissions to seek their support, endorsement and, ... their co-sponsorship."<br><br>Using the 1993 PMI Marketing Code as a	

Study	Geography	What policy is the TI attempting to influence?	Tactics used	Arguments used
			model, the company's Latin American subsidiaries issued codes that promised to "market their cigarettes responsibly and only to adults" to prevent the approval of effective governmental tobacco control regulations. Generally, the codes were endorsed by the local national manufacturer's association, the local advertising agency association, the International Advertising Association, and the media trade association, and were signed by PMI and BAT.<br><br>On September 11, 1997. the Lower House of Paraguay passed a bill to regulate tobacco and alcohol advertising and promotion… As a counterproposal, CERNECO offered to work with the minister of education to continue implementing the tobacco industry's education program "Yo Tengo PO,D.E.R," ("I Have Power") and the retailer program "Es lo Correcto" ("It's the Right Thing").	
Sebrie et al, 2005 [40]	Argentina	General marketing regulation	After securing the Neri Bill veto, the industry started to prepare new legislation as ‘‘a constructive counter-proposal’’ to protect their interests. According to PMLA’s vice president,‘‘[the] tobacco industry maintained frequent contact with Senate Health	

Study	Geography	What policy is the TI attempting to influence?	Tactics used	Arguments used
			Committee to discuss alternative proposals which culminated in the preparation of substitute bills by several senators for consideration by the committee''.<br><br>In November 1992, Senator Pedro Molina, President of the Public Health Committee, introduced an ''alternative bill'' in the Senate that was consistent with the industry's voluntary advertising code.<br><br>The industry also sought to pre-empt meaningful anti-tobacco education with its own ''youth smoking prevention'' programmes. The PMI's five point ''Youth Access Prevention & Education Programs'' (including local marketing codes, sampling guidelines, minimum age of purchase laws, education and retailer programmes) were developed for Latin America by 1997, to shift the focus away from the industry's advertising and marketing (as in other countries).	
Shirane et al, 2012 [41]	Czech Republic	1992 Consumer Protection Act. A further advertising ban was passed in	PM... pursued ''all available means to obtain a favourable amendment''<br><br>It used a previously established organisation, Libertad, which, although fully funded by PM, positioned itself as not-for-profit. Supported	

Study	Geography	What policy is the TI attempting to influence?	Tactics used	Arguments used
		December 1993, and government started working on a new advertising law in March 1994. 1994–1995, the Czech Parliament approved an amendment to the Law of Prevention of Alcoholism and Other Drug Addictions	by the global public relations company Burson-Marsteller, Libertad helped frame freedom to advertise tobacco products as a matter of commercial free speech. The campaign was successful and the advertising ban was formally cancelled in July 1993. PM subsequently worked to produce a voluntary code of conduct, presumably to decrease the likelihood that another legislative ban would be proposed<br><br>However, to PM's apparent surprise, a further advertising ban was passed in December 1993, which PM again worked ''to reverse'', promoting self-regulation as an alternative. In February 1994, a vote on relaxing the ban was passed, allowing existing tobacco advertising contracts to run until December 1994 or until a new law was passed, meaning that, although tobacco advertising was technically banned, it still existed throughout the country. Just days prior to this vote, PM had taken five Czech Members of Parliament (MPs) to a two-day all-expenses paid ''briefing trip'' to Switzerland.<br><br>The government started working on a new advertising law in March 1994, and by April	

Study	Geography	What policy is the TI attempting to influence?	Tactics used	Arguments used
			1994, PM had become directly involved with its development. The new law was approved in October and in line with PM's objective, relied on self-regulation.<br><br>PM documents note that a ''behind the scenes approach'' helped them achieve success. A key component of this approach was the establishment of the Council for Advertising, an organisation made up of advertisers and the media which was charged with administering an industry marketing code, closely modelled on PM's own internal code. At least two documents suggest PM was involved in establishing the Council for Advertising and another suggests PM helped fund it.<br><br>By 1994–1995, the Czech Parliament approved an amendment to the Law of Prevention of Alcoholism and Other Drug Addictions, which included a ban on day-time TV and radio advertising for tobacco products. However, the law was rejected by President Havel, following ''several weeks of intensive lobbying by the industry''.	
Stanton et al, 2009 [42]	USA (Maine)	Public Law 470: bill to regulate	In an email from Kent Wold, RJ Reynolds Office of Government Affairs, to Roger	

Study	Geography	What policy is the TI attempting to influence?	Tactics used	Arguments used
		tobacco displays and sales targeted towards children	Mozingo, Senior Vice President of RJ Reynolds, the tactic was described as requiring a one-time only license for cigarette retailers, which in return, "the industry obtained local government preemption for self service displays and advertising." The bill was passively supported by the Maine Grocers Association and the Maine Retail Merchants Association. The legislation was lobbied by the industry, which argued it was a good alternative to a more restrictive bill.	
Szilágyi and Chapman, 2004 [43]	Hungary	Advertising ban	As early as 1992 PM promoted the establishment of a working group aimed at studying "the issues involved in liberalising the current ad ban" with representatives of the industry, ministries, and the Hungarian association of cigarette producers. In spite of the repeated violation of advertising rules by TTCs – such as with BAT addressing direct mails to children -, high-level government officials decided to commence negotiations with the industry: "Despite the damage done by the BAT's direct main campaign… Deputy Secretary of State Schagrin agreed to go ahead with a December 3rd [1992] meeting to discuss a constructive solution to the problems with interpretation of existing	PM considered the Hungarian tobacco industry "a significant and steady source of government tax revenue if healthy and prosperous" and claimed that the freedom to advertise was essential for the continuous influx of capital to be used for the modernisation of the Hungarian tobacco industry: "A critical factor for the successful implementation of the Philip Morris proposal to modernise the Hungarian tobacco industry is the complete freedom of manufacturers to advertise their products… consumers must be provided with

Study	Geography	What policy is the TI attempting to influence?	Tactics used	Arguments used
			legislation on tobacco advertising”.<br><br>The law governing the advertising ban was administered by the Ministry of Trade and Industry (MTI) with the Health Ministry – always among the weakest portfolios of successive Hungarian governments – also invited to participate in discussions in late 1992. TTCs sought urgent liberalisation of advertising rules and offered a voluntary marketing code; they knew that “if done honestly and with the concurrence of government authorities – and in advance of restrictive government proposals – industry codes can be accepted instead of legislation”.<br><br>The Ministry of Agriculture (MoA) supported PM’s effort in using the working group as a forum “to negotiate the development of a decree which will serve as a more practical interpretation of the Trade Law [one of the regulations on tobacco advertising]”. Members of the working group included the representatives of the Hungarian TTCs, but also delegates of the alcohol industry. The latter was another partner interested in the amendment of the 1978 decree since alcohol	the essential characteristics of the product entries in an impactful way”.

Study	Geography	What policy is the TI attempting to influence?	Tactics used	Arguments used
			advertising was also banned at that time.<br><br>Meanwhile, the Hungarian Association of Cigarette Manufacturers (HACM), soon after its establishment by TTCs in 1994, took up the leadership in orchestrating the development of a voluntary code on advertising. The then operating Hungarian TTCs (PM, BAT, Reemtsma and R.J. Reynolds) put together a more detailed “industry proposal for modernisation of the regulatory environment regarding the marketing of tobacco products”, in an attempt to “reopen discussions with the government”. The voluntary code, “proposed and elaborated by PM”, was integral part of this document….The document package was accepted and signed by representatives of TTCs on 27 October 1994. The MTI was chosen as the “initial avenue” for lobbying since “their legal department drafted the versions A and B of the former law proposal”. In addition, Andreas Gembler of PM Europe had already lubricated the process by discussing the issue with László Pál, then trade minister during the Hungarian Grand Prix (37). The HACM also engaged itself in a lobbying tour promoting a weak law, which allowed enough room for	

Study	Geography	What policy is the TI attempting to influence?	Tactics used	Arguments used
			industry self-regulation: "The Hungarian Association of Cigarette Manufacturers (HACM) gave a presentation on the economic impact of the tobacco industry in Hungary and presented the HACM position on tobacco advertising to representatives of the legal sections on the Ministries of Industry & Trade, Labour, Finance, Culture & Education, and Health. The meeting was successful and will foster further dialogue. In particular, the representative of the Ministry of Health called for a compromise on the two versions of the tobacco advertising law draft, an industry voluntary code and an enforceable regulatory regime on advertising".<br><br>HACM: As well as direct lobbying of ministry officials, MPs and parliamentary committees, it also communicates industry positions in the news media. It commissions studies and economic analyses to support industry views and actively promotes these among key decision makers.<br><br>The Self Regulatory Advertising Board (SRAB), ostensibly established to protect the public from deceptive advertising, seemed to be the	

Study	Geography	What policy is the TI attempting to influence?	Tactics used	Arguments used
			most faithful supporter of the TTCs. The organisation, established on March 1996, just a few months before the launching of the parliamentary debate of the advertising bill, is a vocal supporter of self-regulation, claiming that it provides more effective and flexible solutions to the regulation of the advertising industry itself and to the control of conflicts between the industry and the public. Both BAT and PM were founding members of the organisation, along with advertising agencies with close links to tobacco companies. Moreover, both BAT and PM have had their representatives elected members of SRAB's governing bodies.	
Tsoukalas et al, 2003 [44]	USA (Minnesota)	HF 117 – youth access legislation	A few days after HF 117 was introduced, the tobacco industry put in place a plan to have what it called "a retailer alternative" bill to refer to the House Commerce Committee. The tobacco industry's troops on this were the Minnesota Grocers Association, the Minnesota Retailers Association, and the Minnesota Petroleum Marketers Association. By January 24, 1997, Philip Morris had in place a script for a phone bank to call Minnesota retailers with claims such as "HF 117 could severely restrict your ability to sell tobacco to	

Study	Geography	What policy is the TI attempting to influence?	Tactics used	Arguments used
			your adult customers," and "If this bill becomes law, it could have a negative economic impact on your business". The phone bank surveyor was then directed to urge the tobacco retailers to call their state representative to oppose HF 117 (Philip Morris' phone bank objective was to have the retailers target the entire State House of Representatives). Another tactic Philip Morris used was to have its chief midwest lobbyist, Jack Lenzi, place calls to all House Commerce Committee members using them to oppose HF 117.<br><br>Philip Morris' phone bank sought to encourage retailers to push their legislators to adapt preemptive laws to eliminate local government's ability to enact tougher youth access related ordinances. On February 6, 1997, the Minnesota House passed HF 117 with the preemption exclusion intact	
Tumwine, 2011 [45]	Africa	Advertising ban	Tobacco industry interference is a major obstacle that African countries face in their efforts to ban tobacco advertising, promotion and sponsorship. A formerly secret industry document belonging to BAT revealed that a "proposed ad ban in Sierra Leone, taken out	

Study	Geography	What policy is the TI attempting to influence?	Tactics used	Arguments used
			of the Cabinet at voting stage" was an example of "marketing freedoms in Africa maintained through concerted government relations and effective community involvement programmes"	
Tung and Glantz, 2008 [46]	USA (Nevada)	Senate Bills 622 (TI supported) and 637 (Tobacco Prevention Coalition supported) – legislation to comply with the requirements of the federal Synar amendment (federal requirement for states to pass laws restricting the sale of tobacco to minors)	Senate Bill 622 was drafted and backed by the tobacco industry.<br><br>Senate Bill 622 as originally introduced gave sheriff departments and law enforcement organizations the responsibility for conducting the random inspections of tobacco merchants and reporting of compliance with state law restricting the sale of tobacco products to minors. SB 622 also sought to introduce what would be later be called super preemption, which was legislation preventing local governments from imposing more stringent restrictions on anything related to tobacco, specifically the smoking, use, sale, distribution, marketing, display, or promotion of tobacco products.	
Washington	USA	1998 advertising		In 1988, the three local chapters of

Study	Geography	What policy is the TI attempting to influence?	Tactics used	Arguments used
et al, 2012 [47]	(Kentucky)	restrictions		voluntary agencies, American Cancer, Heart, and Lung joined together to create the Tobacco-Free Young Kentuckians coalition...The Coalition was primarily formed to implement the national tri-agency Smoke-Free Class of 2000 program in Kentucky. The Smoke-Free Class of 2000 program was a 12-year program designed specifically to target children in the first grade in 1988, with a with tobacco prevention, awareness and education, in an effort to prevent youth smoking. The tobacco industry monitored the activities of the national program and used the program activities to develop arguments to oppose advertising restrictions in 1998 by claiming that youth were well aware about the dangers of smoking as a result of the national Smoke-free Class of 2000 program.
Welle et al, 2004 [48]	USA (North Dakota)	Vending machine restrictions	The Tobacco Institute outlined their strategy in a "Pro-Active Proposal" created prior to the beginning of the 1991 legislative session.	Following the standard tobacco industry strategy of predicting that tobacco control measures would

Study	Geography	What policy is the TI attempting to influence?	Tactics used	Arguments used
		(1992)	According to the Tobacco Institute:<br>"The tobacco wholesalers have expressed some interest in pushing for a state-wide preemptive bill".<br><br>Wolf (Tobacco Institute lobbyist) stated that he would be providing Waltman and Carlson (vending machine company owners) with information to use when talking with council members and "alternate language that could be used to seek a compromise with the proponents of the City Commission.	hurt the economy, Dave Schwann, president of Schwann Wholesale (vending machine dealer), Devils Lake, provided loss of business statistics including "56 vending machines would be lost, 21% of the total [Devil's Lake]. $45,000 would be lost in gross profit, and one job would be lost in Devil's Lake."
WHO, 2008 [49]	Middle East (UAE and Lebanon)	1. UAE – 1990 proposed ban on outdoor advertising, promotions and sampling<br>2. Lebanon – 2000 advertising ban	**1**<br>In 1990, the tobacco industry in the United Arab Emirates successfully defeated proposals to ban outdoor advertising, promotions and sampling. "Working within and with the United Arab Emirates Chapter of the IAA, META members with their advertising agencies and suppliers, were able to sensitise key decision makers on the potential implications of the ban …."<br><br>In order to forestall government restrictions on tobacco advertising, the companies devised and promoted voluntary codes on	**1**<br>"Working within and with the United Arab Emirates Chapter of the IAA, META members with their advertising agencies and suppliers, were able to sensitise key decision makers on the potential implications of the ban on local businesses and Dubai's efforts to become a centre of tourism in the GCC. This mobilization under the IAA's umbrella succeeded in suspending the implementation of the proposed ad ban and proved that Industry

Study	Geography	What policy is the TI attempting to influence?	Tactics used	Arguments used
			advertising. The goal of such codes, according to META, was "the goodwill to be gained with the UAE authorities to the benefit of the industry from being able to point to a voluntary 'in-house' document formalizing – in an informal way – standards of behaviour which at an unknown future date the authorities could well enforce anyway"<br><br>**2**<br>A copy of a 20 March 2000 memo from Bisharah Baroudi of Philip Morris to Nicolas Hobeiche details steps that BAT and Philip Morris were taking in reaction to proposals in Lebanon for an advertising ban. Beginning with an "informal approach" by Ramzi Najjar of Bates-Rouge (BAT's advertising agency) to the Minister of Health at a dinner function, the companies have been trying to play on the Minister's concern for youth smoking by proposing joint government-industry cooperation on youth smoking prevention. In the memo, Baroudi proposes "constructive engagement and cooperation with the Ministry of Health in addressing youth smoking", including encouraging minimum	action through the local Chapter of the IAA can be very effective in defending marketing and sponsorship freedoms."

Study	Geography	What policy is the TI attempting to influence?	Tactics used	Arguments used
			age legislation and launching retail access prevention programs.	

Explanation of systematic searches

Search string: (corporat* OR industr* OR compan* OR busines* OR firm*) AND (tobacco OR smok* OR cigarette*) AND (marketing OR advertis* OR sponsor*) AND (regulat* OR policy OR legislat*)

Table 5: Searches completed and the number of articles returned

Location of search	Search notes	Articles returned *(excluding duplicates)*
Web of Knowledge (which included Web of Science, BIOSIS Previews and MEDLINE)	Full string in 'Topic'	672
Business Source Premier	Full string in 'Topic', except '(tobacco OR smok* OR cigarette*)' in 'Title' (searching with it in 'topic' returned far too many hits)	231
EMBASE	Full string in 'Topic'	227
UCSF Tobacco Documents 'Marketing and Advertising' Bibliography (www.library.ucsf.edu/tobacco/docsbiblio/marketing)	Search string could not be applied so all studies published on this site were initially considered based on their title	99
UCSF reports on state tobacco policymaking (www.tobacco.ucsf.edu/states)	Search string could not be applied so all studies published on this site were initially considered based on their title	45
Contacting experts	E-mail	417
Search engine 'Google'	Full string	63
	**Total**	**1754**

References

1. Lord MD (2003) Constituency Building as the Foundation for Corporate Political Strategy. The Academy of Management Executive (1993-2005) 17: 112-124.
2. Alechnowicz K, Chapman S (2004) The Philippine tobacco industry: "the strongest tobacco lobby in Asia". Tobacco Control 13 (Suppl. 2): ii71-78.
3. Apollonio DE, Malone RE (2010) The "We Card" Program: Tobacco Industry "Youth Smoking Prevention" as Industry Self-Preservation. American Journal of Public Health 100: 1188-1201.
4. ASH (2010) The smoke filled room: how big tobacco influences health policy in the UK. Available: www.ash.org.uk/smokefilledroom. Accessed: 04/2011
5. Freeman B, Chapman S, Rimmer M (2008) The case for the plain packaging of tobacco products. Addiction 103: 580-590.
6. Physicians for a Smoke-Free Canada (2008) The Plot Against Plain Packaging: how multinaitonal tobacco companies colluded to use trade arguments they knew were phoney to oppose plain packaging. And how health ministers in Canada and Australia fell for their chicanery. Ottawa, Ontario.
7. Assunta M, Chapman S (2004) A mire of highly subjective and ineffective voluntary guidelines: Tobacco industry efforts to thwart tobacco control in Malaysia. Tobacco Control 13 (Suppl. 2): ii43-ii50.
8. Bond L, Daube M, Chikritzha T (2009) Access to confidential alcohol industry documents: from 'Big Tobacco' to 'Big Booze'. Australasian Medical Journal 2: 1-26.
9. Carter SM (2003) From legitimate consumers to public relations pawns: the tobacco industry and young Australians. Tobacco Control 12 (Suppl. 3): iii71-78.
10. Chapman S, Carter SM (2003) "Avoid health warnings on all tobacco products for just as long as we can": a history of Australian tobacco industry efforts to avoid, delay and dilute health warnings on cigarettes. Tobacco Control 12: iii13-iii22.
11. Cohen JE, Sarabia V, Ashley MJ (2001) Tobacco commerce on the Internet: a threat to comprehensive tobacco control. Tobacco Control 10: 364-367.
12. Dearlove J, Glantz SA (2000) Tobacco Industry Political Influence and Tobacco Policy Making in New York 1983-1999. Available: http://tobacco.ucsf.edu/states. Accessed: 07/2011
13. DiFranza JR, Godshall WT (1996) Tobacco industry efforts hindering enforcement of the ban on tobacco sales to minors: actions speak louder than words. Tobacco Control 5: 127-131.
14. Epps-Johnson T, Barnes R, Glantz SA (2009) The Stars Aligned Over the Cornfields: Tobacco Industry Political Influence and Tobacco Policy Making in Iowa 1897-2009. Available: http://tobacco.ucsf.edu/states. Accessed: 07/2011
15. Gilmore AB, Collin J, McKee M (2006) Public health: British American Tobacco's erosion of health legislation in Uzbekistan. BMJ 332: 355.
16. Goldberg ME, Davis RM, O'Keefe AM (2006) The role of tobacco advertising and promotion: themes employed in litigation by tobacco industry witnesses. Tobacco Control 15: 54-67.

17. Hammond D (2009) Tobacco labelling and packaging toolkit: a guide to FCTC Article 11, 2009. Available: www.tobaccofreeunion.org/files/186.pdf. Accessed: 06/2011
18. Hammond D (2010) "Plain packaging" regulations for tobacco products: the impact of standardizing the color and design of cigarette packs. Salud Pública de México 52: S226-S232.
19. Hiilamo H, Crosbie E, Glantz SA (2012) The evolution of health warning labels on cigarette packs: the role of precedents, and tobacco industry strategies to block diffusion. Tobacco Control online first.
20. Hoek J, Vaudrey R, Gendall P, Edwards R, Thomson G (2012) Tobacco retail displays: a comparison of industry arguments and retailers' experiences. Tobacco Control 21: 497-501.
21. Ibrahim JK, Glantz SA (2003) Tobacco Policy Making in California 2001-2003: No Longer Finishing First. Available: http://tobacco.ucsf.edu/states. Accessed: 07/2011
22. Kennedy A, Sullivan S, Hendlin Y, Barnes R, Glantz SA (2011) Tobacco Control in Florida 1999-2011: The Good, The Bad, and The Ugly. Available: http://tobacco.ucsf.edu/states. Accessed: 07/2011
23. Lambert A, Sargent JD, Glantz SA, Ling PM (2004) How Philip Morris unlocked the Japanese cigarette market: lessons for global tobacco control. Tobacco Control 13: 379-387.
24. Landman A, Ling PM, Glantz SA (2002) Tobacco Industry Youth Smoking Prevention Programs: Protecting the Industry and Hurting Tobacco Control. American Journal of Public Health 92: 917-930.
25. Lee C, Glantz S (2001) The Tobacco Industry's Successful Efforts to Control Tobacco Policy Making in Switzerland. Available: www.who.int/tobacco/media/en/InquirySwiss.pdf. Accessed: 05/2011
26. Ling PM, Haber LA, Wedl S (2010) Branding the Rodeo: A Case Study of Tobacco Sports Sponsorship. American Journal of Public Health 100: 32-41.
27. MacKenzie R, Collin J, Sopharo C, Sopheap Y (2004) "Almost a role model of what we would like to do everywhere": British American Tobacco in Cambodia. Tobacco Control 13 (Suppl. 2): ii112-117.
28. Mackey TK, Liang BA, Novotny TE (2013) Evolution of Tobacco Labeling and Packaging: International Legal Considerations and Health Governance. American Journal of Public Health 103 (4).
29. Mamudu HM, Hammond R, Glantz SA (2008) Project Cerberus: Tobacco industry strategy to create an alternative to the Framework Convention on Tobacco Control. American Journal of Public Health 98: 1630-1642.
30. Mandal S, Gilmore A, Collin J, Weishaar H, Smith K, et al. (2009) Block, amend, delay: tobacco industry efforts to influence the European Union's Tobacco Products Directive (2001/37/EC). Available: http://www.smokefreepartnership.eu/news/block-amend-delay-tobacco-

industry-efforts-influence-european-union%E2%80%99s-tobacco-products. Accessed: 06/2011
31. McDaniel PA, Malone R (2005) Understanding Philip Morris's pursuit of US government regulation of tobacco. Tobacco Control 14.
32. McDaniel PA, Malone RE (2009) Creating the "Desired Mindset": Philip Morris's Efforts to Improve Its Corporate Image Among Women. Women & Health 49: 441-474.
33. Mekemson C, Glantz SA (2002) How the tobacco industry built its relationship with Hollywood. Tobacco Control 11: I81-I91.
34. Nakkash R, Lee K (2009) The tobacco industry's thwarting of marketing restrictions and health warnings in Lebanon. Tobacco Control 18: 310-316.
35. Neuman M, Bitton A, Glantz S (2002) Tobacco industry strategies for influencing European Community tobacco advertising legislation. Lancet 359: 1323-1330.
36. O'Sullivan B, Chapman S (2000) Eyes on the prize: transnational tobacco companies in China 1976-1997. Tobacco Control 9: 292-302.
37. Outterson K (2011) Smoking and the First Amendment. New England Journal of Medicine 365: 2351-2353.
38. Pan American Health Organisation (2002) Profits Over People: Tobacco Industry Activities to Market Cigarettes and Undermine Public Health in Latin America and the Caribbean.
39. Sebrie EM, Glantz SA (2007) Tobacco industry "Youth smoking prevention" programs to undermine meaningful tobacco control in Latin America. American Journal of Public Health 97: 1357-1367.
40. Sebrie EM, Barnoya J, Perez-Stable EJ, Glantz SA (2005) Tobacco industry successfully prevented tobacco control legislation in Argentina. Tobacco Control 14.
41. Shirane R, Smith K, Ross H, Silver KE, Williams S, et al. (2012) Tobacco Industry Manipulation of Tobacco Excise and Tobacco Advertising Policies in the Czech Republic: An Analysis of Tobacco Industry Documents. PLoS Medicine 9: e1001248.
42. Stanton C, Barnes R, Glantz SA (2009) Tobacco Control in Maine, 1979-2009: The Power of Strategic Collaboration. Available: http://tobacco.ucsf.edu/states. Accessed: 07/2011
43. Szilagyi T, Chapman S (2004) Tobacco industry efforts to erode tobacco advertising controls in Hungary. Central European Journal of Public Health 12: 190-196.
44. Tsoukalas TH, Ibrahim JK, Glantz SA (2003) Shifting Tides: Minnesota Tobacco Politics. Available: http://tobacco.ucsf.edu/states. Accessed: 07/2011
45. Tumwine J (2011) Implementation of the Framework Convention on Tobacco Control in Africa: Current Status of Legislation. International Journal of Environmental Research and Public Health 8: 4312-4331.
46. Tung G, Glantz SA (2008) Swimming Upstream: Tobacco Policy Making in Nevada. Available: http://tobacco.ucsf.edu/states. Accessed: 07/2011

47. Washington MD, Barnes RL, Glantz SA (2012) Good Start Out of the Gate: Tobacco Industry Political Influence and Tobacco Policymaking in Kentucky 1936-2012. Available: http://tobacco.ucsf.edu/states. Accessed: 07/2011
48. Welle JR, Ibrahim JK, Glantz SA (2004) Tobacco Control Policy Making in North Dakota: A Tradition of Activism. Available: http://tobacco.ucsf.edu/states. Accessed: 07/2011
49. WHO (2008) Voice of Truth. Egypt: WHO Regional Office for the Eastern Mediterranean.
