# Supplementary material for: How Does the Tobacco Industry Attempt to Influence Marketing Regulations? A Systematic Review
Source: PLoS One. 2014 Feb 5;9(2):e87389. doi: 10.1371/journal.pone.0087389 (PMC3914831; doi:10.1371/journal.pone.0087389)
Supplement: Flow Diagram S1 — (PDF) [file pone.0087389.s003.pdf]

PRISMA 2009 Flow Diagram

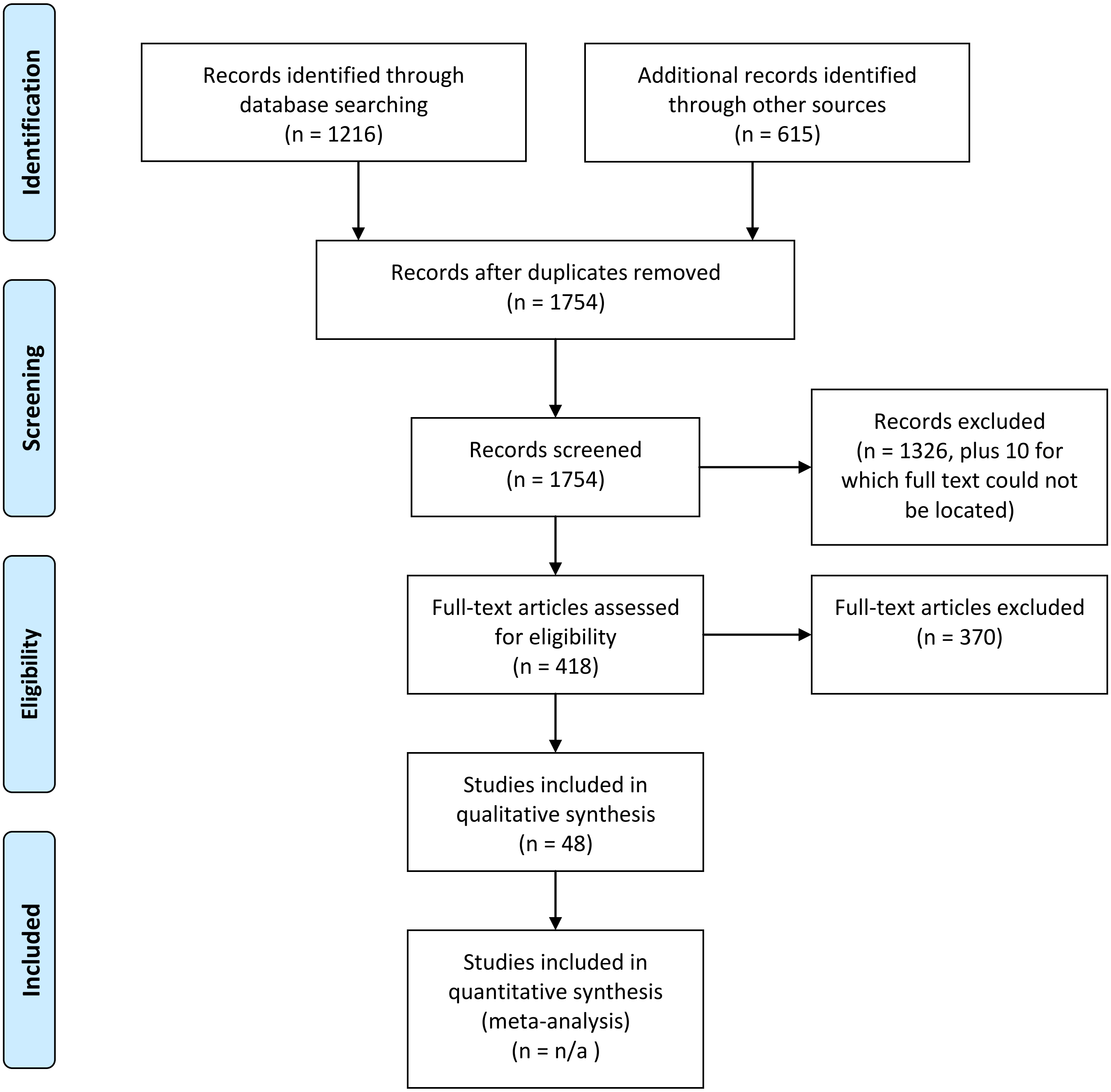

From: Moher D, Liberati A, Tetzlaff J, Altman DG, The PRISMA Group (2009). *P*referred *R*eporting *I*tems for *S*ystematic Reviews and *M*eta-*A*nalyses: The PRISMA Statement. PLoS Med 6(6): e1000097. doi:10.1371/journal.pmed1000097

For more information, visit www.prisma-statement.org.
