# Supplementary material for: How Does the Tobacco Industry Attempt to Influence Marketing Regulations? A Systematic Review
Source: PLoS One. 2014 Feb 5;9(2):e87389. doi: 10.1371/journal.pone.0087389 (PMC3914831; doi:10.1371/journal.pone.0087389)
Supplement: Protocol S1 — (PDF) [file pone.0087389.s004.pdf]

SYSTEMATIC REVIEW PROTOCOL

Title

How does the tobacco industry attempt to influence marketing regulations? A systematic review

Rationale

Marketing is the main way in which corporations communicate with their current and potential customers and consumers, and it encompasses five key variables: product, promotion, price, place, and person[1]. Despite tobacco industry (TI) claims that their marketing is only used for brand switching and capturing market share, existing research clearly shows that there is a significant link between TI marketing and smoking initiation amongst young people and increased smoking prevalence[2,3,4,5,6,7,8]. This underpins the importance of understanding the strategies the TI use to shape policies aimed at regulating the marketing of tobacco products which kill half of their long-term users[9,10].

The public availability of internal TI documents resulting from state-level litigation and the signing of the Master Settlement Agreement in the USA has formed the basis of an extensive body of work on TI political activity (see [11] for overview), however studies tend to be event or case-study based. Although providing potentially valuable detail of the political strategies used by the TI, they do not draw out the broader trends and patterns of TI political activity. To date only two studies have reviewed elements of this literature systematically[12,13], and none have attempted to develop taxonomies where industry tactics and arguments can be assessed and systematically categorised in a way that could be applied to other areas of public health involving corporate interests.

This systematic review therefore aims to both systematically review the strategies used by the TI to influence regulation aimed at restricting the marketing of tobacco products, and to develop taxonomies for categorising the tactics and arguments used.

By providing a summary of TI actions, this review is likely to be a valuable resource for enhancing the ability of public health advocates and policymakers to understand, predict, and potentially counter tactics the TI might use to exert influence on policy and the types of arguments it is most likely to make when it does. Multiple Articles of the World Health Organisation's Framework Convention on Tobacco Control (FCTC) make recommendations regarding the marketing of tobacco products. The FCTC covers 87.4% of the world's population[14], but despite the vast majority of states becoming

Party to the FCTC many have yet to implement its recommendations[15] with the tactics of the TI identified as a hindrance to the development and implementation of legislation[16].

Review question and aims

This review seeks to establish, through the available literature, an answer to the following question:

How has the tobacco industry attempted to influence regulation aimed at restricting the marketing of tobacco products from 1990 to the present day?

The review has two key aims:

1. To systematically review the strategies used by the TI to influence regulation aimed at restricting the marketing of tobacco products

2. To develop taxonomies of:
    a. industry strategies and tactics
    b. industry frames and arguments

Inclusion/exclusion criteria

To be included in this review, studies and individual tactics and arguments must fulfil the following criteria:

- Studies must be written in English.
- Studies must cover the period from 1990-2013. In papers that cover both before and after 1990, only those tactics/arguments relating to post-1990 will be recorded and included within this review.
- Studies must look at TI efforts to influence new regulatory measures regarding marketing regulation/policy (information regarding how the industry attempts to circumvent existing regulation will not be included within the review).
- The tactics/arguments covered must be related to one or more of the following: product (for example, packaging, new products/flavours, branding), price* (for example, price promotions, minimum pricing), promotion (advertising including billboards, point-of-sale, sponsorship), place (for example, vending machines, restrictions on advertising near schools) or person (for example, restrictions on advertising or selling to youth).

- Each individual claim made regarding TI tactics/arguments used to influence marketing regulation must be directly supported by verifiable evidence.
- Tactics/arguments identified must be directly implemented by the TI or by a group where substantial evidence suggests that they act on the TI's behalf.
- Tactics/arguments which are noted within the included articles are assumed to have been carried through, in the absence of evidence to the contrary. Tactics/arguments which are shown to only have been planned, and not used, will not be recorded.
- Only tactics/arguments directly related to marketing regulation will be recorded. For example, health warning labels are included as they influence the means of packaging as a marketing tool, but they are excluded if the study only looks at, for example, the wording of the warning, as this does not affect marketing.
- Only tactics/arguments that are clearly detailed in the paper(s) are coded.

* Price in the form of tax will be excluded as a separate review on tax-related lobbying has already been completed (see [12]) and this would therefore overlap. Price in terms of price-based promotions will be included.

Search strategy

In order to reduce the chance of missing any relevant articles, both automated and manual searches will be completed.

Automated searches will be completed using the following key terms (following the advice of a qualified librarian):

- Corporat*, Industr*, Compan*, Busines*, Firm*
- Tobacco, Smok*, Cigarette*
- Marketing, Advertis*, Sponsor*
- Regulat*, Policy, Legislat*

Searches using these terms will be undertaken in Web of Knowledge (which includes Web of Science, BIOSIS Previews, and MEDLINE), Business Source Premier, and Embase (databases selected following the advice of a qualified librarian). The search engine Google will be used to identify grey literature, the UCSF Tobacco Documents 'Marketing and Advertising' Bibliography[17] will be

searched for additional academic articles, the series of UCSF US State tobacco reports[18] will be assessed, and experts will be contacted to identify any additional papers.

All searches, and subsequent article retrieval, will be limited to articles published since 1990, and articles written in English.

Identified articles will first be screened for potential relevance by their title and, if available, their abstract. Articles that appear relevant to the study question will be downloaded for full analysis, and will then be carefully checked against the detailed inclusion/exclusion criteria (above). If articles do not meet all of the criteria they will be excluded. The remaining articles will be included within the systematic review.

Data extraction

After checking each article against the inclusion criteria, those that meet all of the criteria will be subjected to data extraction. The table used to review each article can be seen below. Data extraction of included articles will initially be undertaken by the lead author, and a sample will be second-reviewed by both the second and third authors to check that all of the inclusion criteria have been met. If there are any discrepancies, additional articles will be second-reviewed until all authors are certain that all articles meet the criteria.

The tactics and arguments identified through data extraction will then be categorised and counted. This review will split TI political activity into 'strategies' which include individual 'tactics' (the methods by which a corporation attempts to exert influence) and 'frames' which include individual 'arguments' (the reasons given by a corporation as to why they oppose one idea or support another).

The list of strategies/tactics and frames/arguments will initially be developed via 'a priori coding'[19], the former adapted from Hillman and Hitt[20] and the latter based on the limited existing literature on TI frames[21,22]. Additional categories will then be added via 'emergent coding'[19] following review of the papers included and after discussion between all three authors. The taxonomies will only be finalised after all of the papers have been reviewed. Any differences in tactic/argument categorisation will be discussed between all three authors until agreement has been reached, and all evidence falling under that particular category will be re-reviewed by all three authors to check for consistency.

If a tactic or argument is referred to more than once (in one or multiple articles) regarding the *same* policy then it will only be counted once, whereas if it is referred to more than once about *different* policies then this will be counted separately.

This review is concerned with the quality of evidence for individual tactics and arguments, and not the overall quality of the articles included in the review. The requirement that all tactics and arguments are supported by verifiable evidence (as outlined in the inclusion/exclusion criteria, above) ensures that only high quality evidence will be included in the review.

The evidence will be qualitative, so narrative synthesis will be used to combine the results.

Data extraction form:

**Full reference**				
Reviewer				
**Check list for inclusion**				
Does the study relate to tobacco industry arguments/tactics used to influence marketing regulation (price (not tobacco tax), product, promotion, place, person)?	Yes		No (exclude from review)	
Is the article in English?	Yes, in English		No, not English (exclude from review)	
Is the study concerned with activities which take place from 1990 onwards? (If only concerned with pre-1990 period, exclude; if concerned with before and after 1990, include and note claims from review period).	Yes, study is concerned with post-1990 period (state which period it focuses on).		No, study is only concerned with period before 1990 (exclude from review)	
Are any claims made about tobacco industry arguments/tactics used to influence marketing regulation supported by verifiable evidence? (Only note claims that are supported by verifiable evidence)	Yes		No (exclude from review)	
**Data Extraction**				
What country/region does the article focus on?				
Which companies are studied?				

What are the aims of the study?	
Which policy is industry attempting to influence?	
What tactics does industry use?	
Which arguments does industry make?	
Was industry successful? What was the result?	

References

1. Ennew CT (1993) The Marketing Blueprint. Oxford: Blackwell Business.
2. NCI (2008) The role of the media in promoting and reducing tobacco use. Bethesda, MD: US Department of Health and Human Services: National Institutes of Health. 4302 p.
3. DiFranza JR, Wellman RJ, Sargent JD, Weitzman M, Hipple BJ, et al. (2006) Tobacco promotion and the initiation of tobacco use: assessing the evidence for causality. Pediatrics 117: e1237-e1248.
4. Slater SJ, Chaloupka FJ, Wakefield M, Johnston LD, O'Malley PM (2007) The impact of retail cigarette marketing practices on youth smoking uptake. Archives of Pediatrics & Adolescent Medicine 161: 440.
5. Weiss JW, Cen S, Schuster DV, Unger JB, Johnson CA, et al. (2006) Longitudinal effects of pro-tobacco and anti-tobacco messages on adolescent smoking susceptibility. Nicotine & Tobacco Research 8: 455-465.
6. Pucci LG, Siegel M (1999) Exposure to brand-specific cigarette advertising in magazines and its impact on youth smoking. Preventive Medicine 29: 313-320.
7. Pierce JP, Choi WS, Gilpin EA, Farkas AJ, Berry CC (1998) Tobacco industry promotion of cigarettes and adolescent smoking. JAMA: The Journal of the American Medical Association 279: 511-515.
8. Schooler C, Feighery E, Flora JA (1996) Seventh graders' self-reported exposure to cigarette marketing and its relationship to their smoking behavior. American Journal of Public Health 86: 1216-1221.
9. WHO (2011) Factsheet: Tobacco. Available: http://www.who.int/mediacentre/factsheets/fs339/en/index.html. Accessed: 21/01/2012
10. ASH (2012) Factsheet: Smoking statistics. Available: http://www.ash.org.uk/files/documents/ASH_93.pdf. Accessed: 21/01/2012
11. Hurt RD, Ebbert JO, Muggli ME, Lockhart NJ, Robertson CR (2009) Open doorway to truth: legacy of the Minnesota tobacco trial. Mayo Clinic Proceedings 84 (5): 446-456.
12. Smith KE, Savell E, Gilmore AB (2013) What is known about tobacco industry efforts to influence tobacco tax? A systematic review of empirical studies. Tobacco Control 22.
13. Lee S, Ling PM, Glantz SA (2012) The vector of the tobacco epidemic: tobacco industry practices in low and middle-income countries. Cancer Causes and Control: 1-13.
14. Eriksen M, Mackay J, Ross H (2012) The Tobacco Atlas, 4th ed. Atlanta, Georgia, USA: American Cancer Society, Inc.
15. WHO (2012) FCTC: Reporting on the implementation of the Convention database. Available: http://apps.who.int/fctc/reporting/database/. Accessed: 24/10/12
16. WHO (2010) 2010 Global Progress Report on the Implementation of the WHO Framework Convention on Tobacco Control.; Available: http://www.who.int/fctc/reporting/summaryreport.pdf. Accessed: 14/01/13
17. UCSF Library (2010) Legacy Tobacco Documents Library: Marketing and Advertising. Available: http://www.library.ucsf.edu/tobacco/docsbiblio/marketing. Accessed: 06/2010
18. Glantz S (2010) Reports on State Tobacco Policy Making. Available: http://tobacco.ucsf.edu/states. Accessed: 07/2011
19. Stemler S (2001) An overview of content analysis. Practical Assessment, Research & Evaluation 7: 137-146.
20. Hillman AJ, Hitt MA (1999) Corporate political strategy formulation: A model of approach, participation, and strategy decisions. Academy of Management Review: 825-842.
21. Harris JK, Shelton SC, Moreland-Russell S, Luke DA (2010) Tobacco coverage in print media: the use of timing and themes by tobacco control supporters and opposition before a failed tobacco tax initiative. Tobacco Control 19: 37-43.

22. Champion D, Chapman S (2005) Framing pub smoking bans: an analysis of Australian print news media coverage, March 1996–March 2003. Journal of Epidemiology and Community Health 59: 679-684.
