# Supplementary material for: How Does the Tobacco Industry Attempt to Influence Marketing Regulations? A Systematic Review
Source: PLoS One. 2014 Feb 5;9(2):e87389. doi: 10.1371/journal.pone.0087389 (PMC3914831; doi:10.1371/journal.pone.0087389)
Supplement: Box S1 — (PDF) [file pone.0087389.s005.pdf]

Box S1

Inclusion and Exclusion Criteria

To be included in this review, studies and individual tactics and arguments had to fulfil the following criteria:

- Studies must be written in English.
- Studies must cover the period from 1990-2013. In papers that cover both before and after 1990, only those tactics/arguments relating to post-1990 will be recorded and included within this review.
- Studies must look at TI efforts to influence new regulatory measures regarding marketing regulation/policy (information regarding how the industry attempts to circumvent existing regulation will not be included within the review).
- The tactics/arguments covered must be related to one or more of the following: product (for example, packaging, new products/flavours, branding), price* (for example, price promotions, minimum pricing), promotion (advertising including billboards, point-of-sale, sponsorship), place (for example, vending machines, restrictions on advertising near schools) or person (for example, restrictions on advertising or selling to youth).
- Each individual claim made regarding TI tactics/arguments used to influence marketing regulation must be directly supported by verifiable evidence.
- Tactics/arguments identified must be directly implemented by the TI or by a group where substantial evidence suggests that they act on the TI's behalf.
- Tactics/arguments which are noted within the included articles are assumed to have been carried through, in the absence of evidence to the contrary. Tactics/arguments which are shown to only have been planned, and not used, will not be recorded.
- Only tactics/arguments directly related to marketing regulation will be recorded. For example, health warning labels are included as they influence the means of packaging as a marketing tool, but they are excluded if the study only looks at, for example, the wording of the warning, as this does not affect marketing.
- Only tactics/arguments that are clearly detailed in the paper(s) are coded.

* Price in the form of tax has been excluded as a separate review on tax-related lobbying has already been completed (see [1]) and this would therefore overlap. Price in terms of price-based promotions have been included.
